# Supplementary material for: Drug Use in Street Sex worKers (DUSSK) study: results of a mixed methods feasibility study of a complex intervention to reduce illicit drug use in drug dependent female sex workers
Source: BMJ Open. 2020 Dec 15;10(12):e036491. doi: 10.1136/bmjopen-2019-036491 (PMC7745311; doi:10.1136/bmjopen-2019-036491)
Supplement: Author's manuscript [file bmjopen-2019-036491.draft_revisions.pdf]

BMJ Open

BMJ Open is committed to open peer review. As part of this commitment we make the peer review history of every article we publish publicly available.

When an article is published we post the peer reviewers' comments and the authors' responses online. We also post the versions of the paper that were used during peer review. These are the versions that the peer review comments apply to.

The versions of the paper that follow are the versions that were submitted during the peer review process. They are not the versions of record or the final published versions. They should not be cited or distributed as the published version of this manuscript.

BMJ Open is an open access journal and the full, final, typeset and author-corrected version of record of the manuscript is available on our site with no access controls, subscription charges or pay-per-view fees (http://bmjopen.bmj.com).

If you have any questions on BMJ Open's open peer review process please email info.bmjopen@bmj.com

BMJ Open

Drug Use in Street Sex worKers (DUSSK) study – results of a mixed methods feasibility study of a complex intervention to reduce illicit drug use in drug dependent female sex workers

Journal:	*BMJ Open*
Manuscript ID	bmjopen-2019-036491
Article Type:	Original research
Date Submitted by the Author:	17-Dec-2019

Complete List of Authors:	Patel, Rita; University Hospitals Bristol NHS Foundation Trust, NIHR ARC West; University of Bristol, Population Health Sciences<br>Redmond, Niamh; University Hospitals Bristol NHS Foundation Trust, NIHR ARC West; University of Bristol, Population Health Sciences<br>Kesten, Joanna; University Hospitals Bristol NHS Foundation Trust, NIHR ARC West; University of Bristol, Population Health Sciences & NIHR Health Protection Research Unit in Evaluation of Interventions<br>Linton, Myles-Jay; University Hospitals Bristol NHS Foundation Trust, NIHR ARC West; University of Bristol, Population Health Sciences & Health Economics Bristol<br>Horwood, Jeremy; University Hospitals Bristol NHS Foundation Trust, NIHR ARC West; University of Bristol, Population Health Sciences & Centre for Academic Primary Care<br>Wilcox, David ; Avon & Wiltshire Partnership NHS Trust, Acer Unit, Blackberry Hill Hospital, Manor Road,<br>Munafo , Jessica ; Avon & Wiltshire Partnership NHS Trust, Acer Unit, Blackberry Hill Hospital, Manor Road,<br>Coast, Joanna; University Hospitals Bristol NHS Foundation Trust, NIHR ARC West; Bristol University, Population Health Sciences & Health Economics Bristol<br>Macleod, John; University Hospitals Bristol NHS Foundation Trust, NIHR ARC West; University of Bristol, Population Health Sciences & Centre for Academic Primary Care<br>Jeal, N; University Hospitals Bristol NHS Foundation Trust, NIHR ARC West; University of Bristol, Population Health Sciences

Keywords:	QUALITATIVE RESEARCH, MENTAL HEALTH, HEALTH ECONOMICS

SCHOLARONE™
Manuscripts

I, the Submitting Author has the right to grant and does grant on behalf of all authors of the Work (as defined in the below author licence), an exclusive licence and/or a non-exclusive licence for contributions from authors who are: i) UK Crown employees; ii) where BMJ has agreed a CC-BY licence shall apply, and/or iii) in accordance with the terms applicable for US Federal Government officers or employees acting as part of their official duties; on a worldwide, perpetual, irrevocable, royalty-free basis to BMJ Publishing Group Ltd ("BMJ") its licensees and where the relevant Journal is co-owned by BMJ to the co-owners of the Journal, to publish the Work in this journal and any other BMJ products and to exploit all rights, as set out in our licence.

The Submitting Author accepts and understands that any supply made under these terms is made by BMJ to the Submitting Author unless you are acting as an employee on behalf of your employer or a postgraduate student of an affiliated institution which is paying any applicable article publishing charge ("APC") for Open Access articles. Where the Submitting Author wishes to make the Work available on an Open Access basis (and intends to pay the relevant APC), the terms of reuse of such Open Access shall be governed by a Creative Commons licence – details of these licences and which Creative Commons licence will apply to this Work are set out in our licence referred to above.

Other than as permitted in any relevant BMJ Author's Self Archiving Policies, I confirm this Work has not been accepted for publication elsewhere, is not being considered for publication elsewhere and does not duplicate material already published. I confirm all authors consent to publication of this Work and authorise the granting of this licence.

For peer review only

Drug Use in Street Sex worKers (DUSSK) study – results of a mixed methods feasibility study of a complex intervention to reduce illicit drug use in drug dependent female sex workers

Authors:

Dr Rita Patel*,[1,2] Dr Niamh M Redmond*,[1,2] Dr Joanna M Kesten,[1,2,3] Dr Myles-Jay Linton, [1,2,4] Dr Jeremy Horwood,[1,2,6] David Wilcox,[5] Jess Munafo,[5] Prof Joanna Coast,[1,2,4] Prof John Macleod,[1,2,6] Dr Nicola Jeal.[1,2]

*Joint first authors.

Author affiliations:

1 The National Institute for Health Research Applied Research Collaboration West (NIHR ARC West) at University Hospitals Bristol NHS Foundation Trust, Whitefriars, Lewins Mead, Bristol, UK, BS1 2NT

2 Population Health Sciences, Bristol Medical School, University of Bristol, Canynge Hall, 39 Whatley Road, Bristol, UK, BS8 2PS

3 The National Institute for Health Research Health Protection Research Unit in Evaluation of Interventions, University of Bristol, UK

4 Health Economics Bristol, Population Health Sciences, Bristol Medical School, University of Bristol, 1-5 Whiteladies Road, Bristol, UK, BS8 1NU

5 Avon & Wiltshire Partnership NHS Trust, Acer Unit, Blackberry Hill Hospital, Manor Road, Bristol, UK, BS16 2EW

6 Centre for Academic Primary Care, Population Health Sciences, Bristol Medical School, University of Bristol, Canynge Hall, 39 Whatley Road, Bristol BS8 2PS

Corresponding author:

Dr Nicola Jeal (Nikki.Jeal@bristol.ac.uk)

Word count:

Target Journal BMJ Open: Abstract: (299/300), Main: (3989/4000)

For peer review only

Abstract

Objectives

The majority of female street-based sex workers (SSWs) are dependent on illicit drugs and sell sex to fund their drug use. They typically face multiple traumatic experiences, starting at a young age, which are ongoing through sex work involvement. Their trauma-related symptoms tend to increase when drug use is reduced suggesting a trauma-focussed approach to reducing drug dependency may be effective. Aims to (i) evaluate recruitment and retention of participants; (ii) examine intervention experiences and acceptability; and (iii) explore intervention costs using mixed methods feasibility study.

Setting

Female SSW charity premises in a large UK inner city.

Participants

Females aged 18 years or older, who have sold sex on the street and used heroin and/or crack cocaine at least once a week in the last calendar month.

Intervention

Female SSW-only drug treatment groups in a female SSW-only setting delivered by female staff. Targeted post-traumatic stress disorder (PTSD) screening then treatment of positive diagnoses with eye movement desensitization and reprocessing (EMDR) therapy by female staff from a specialist NHS trauma service.

Results

(i) Of 125 potential participants 11 met inclusion criteria and provided informed consent, 4 for trauma therapy; 2 received EMDR therapy. (ii) Service providers said working in collaboration with other services was valuable, the intervention was worthwhile and had a positive influence on participants. Participants viewed recruitment as acceptable and experienced the intervention positively. The unsettled nature of participant's lives was a key attendance barrier. (iii) The total cost of the intervention was £11,710, with staff costs dominating.

Conclusions

Recruitment rates were lower than anticipated, due to study inclusion criteria targeting those with the most complex needs. Two participants received EMDR demonstrating that three differing agencies

For peer review only

For peer review only - http://bmjopen.bmj.com/site/about/guidelines.xhtml

working together was feasible. Staff heavy costs highlight the important role of supporting participant attendance in minimising per participant intervention cost.

Ethics approval

South West - Frenchay Research Ethics Committee (REC reference: 17/SW/0033; IRAS project ID: 220631). UK HRA approval was on 03/04/2017.The University of Bristol Research Enterprise and Development department sponsored this study (RED reference: RG2756).

Strengths and limitations of this study

- All aspects of the intervention were delivered by specialists
- PPI formed an important part of the study and informed each stage
- Recruitment over several months within a trusted agency with multiple approaches allowed SSWs many opportunities to take part
- This feasibility study provides only preliminary information on the intervention performance and cost and does not examine the effectiveness
- Delays are likely to have adversely influenced study recruitment and retention, and higher service costs

Keywords:

sex workers, feasibility study, qualitative research, cost analysis, trauma treatment, drug dependency

For peer review only - http://bmjopen.bmj.com/site/about/guidelines.xhtml

Introduction

Most female street-based sex workers (SSWs) in the UK use heroin and/or crack cocaine.[1-3] Their drug dependency keeps them entrenched in a "work-score-use cycle",[4 5] which contributes to the morbidity and social instability typically seen in this group.[6]

Despite their drug treatment needs, drug dependent SSWs have poorer outcomes from drug treatment services compared to other service users,[7 8] sometimes due to stigma associated with their street sex work.[9] Previous SSW-focussed interventions aiming to reduce drug use have used educational,[10 11] substitute prescribing-based[12 13] and psychological[14] approaches but none robustly demonstrated a positive effect in reducing drug use.[15]

Poor mental health is a significant problem among SSWs.[16-18] Many have experienced multiple adversities in early life and during their involvement in sex work,[5 16 19] which exposes women to further risk of significant trauma.[16 17] Consequently, many SSWs are affected by post-traumatic stress disorder (PTSD).[16 17] Trauma symptoms, which often recur when drug use is reduced, may motivate a return to drug use.[20] Individual trauma-focused therapy alongside drug treatment may provide the best outcomes for reductions in drug use.[21-23] However to date, there is no robust evidence to demonstrate the impact of an integrated trauma-focussed treatment approach in reducing drug use amongst female drug dependent SSWs.

In collaboration with SSWs and service providers we developed a novel intervention, informed by existing research[9 15] to address the unique and complex combination of drug use and mental health needs of female drug-dependent SSWs. The intervention proposes an integrated care pathway through an innovative multi-agency partnership.[24] We report here the results of the DUSSK (Drug Use in Street Sex worKers) feasibility study, which aimed to (i) evaluate the recruitment and retention of SSWs to the intervention; (ii) examine the experience and acceptability of the intervention for participants and service providers; and (iii) explore costs to service providers associated with the intervention.

For peer review only - http://bmjopen.bmj.com/site/about/guidelines.xhtml

Methods

Study design, setting and eligibility

This mixed methods feasibility study took place in a UK inner city setting. Females aged 18 years or older, who sold sex on the street and used heroin and/or crack cocaine at least once a week in the last calendar month, were eligible to participate.[24] The intervention was delivered at SSW charity premises.

Recruitment

Local organisations that SSWs were known to access were provided with study promotional materials. One of three researchers (JMK, NJ, SR) attended an SSW support and advocacy charity, at least twice a week, to directly approach potential participants; alternatively, interested SSWs could telephone researchers. Eligibility screening was conducted face-to-face or via telephone. Women gave fully informed, written consent to participate in the study at the time of recruitment and baseline data were collected. To maintain safety and confidentiality, each participant provided details of acceptable ways in which to be contacted. Screening data were retained and remained anonymised for those not recruited.

Patient and Public Involvement (PPI)

Women with experience of street sex work and drug-dependency contributed to the study design, processes, documentation and intervention development. Recommendations from PPI meetings were implemented and resulted in direct changes to the study processes to support recruitment, participation and encourage adherence (described below).

The intervention

The intervention consisted of SSW only drug treatment groups, targeted screening for PTSD and, if positively diagnosed, one-to-one EMDR therapy, all delivered by female staff through a collaboration between three service providers (National Health Service (NHS) trauma services, the SSW and drug treatment charities). The intervention was designed so participants were initially invited to attend a weekly 'Getting started' drug treatment group to reduce fear and anxiety about engaging in a group setting and get used to the format and level of disclosure expected. Participants were to progress to

For peer review only

For peer review only - http://bmjopen.bmj.com/site/about/guidelines.xhtml

a ‘Preparation for recovery’ drug treatment group which focused on peoples’ barriers to motivation for change, examining pros and cons of drug use and exploring triggers for using drugs to enable participants to achieve a level of drug use stability. When group facilitators judged participants were achieving drug use stabilisation, and they had attended three sessions consecutively, they were offered screening for PTSD by a female clinical psychologist (one-to-one clinical interview and PTSD checklist- PCL5). [25] Those experiencing PTSD symptoms were invited to attend five PTSD ‘Stabilisation’ group sessions, facilitated by the same female clinical psychologist, to equip participants with the skills to self-soothe and reorientate in preparation for the one-to-one EMDR treatment. Once all ‘Stabilisation’ group sessions had been completed, the clinical psychologist assessed participants for readiness for one-to-one EMDR sessions and if eligible, participants progressed to a course of 12 sessions with the clinical psychologist on a weekly, or fortnightly, basis. Trauma treatment (PTSD screening, stabilisation groups and one-to-one) ran in parallel to the drug treatment groups, Figure 1 (red boxes) shows the planned flow of participants through the study. The intervention proceeded as described[24] with the following changes (bulleted below):

- Participants were encouraged to attend all sessions with the offer of car lifts, bus tickets and taxis, in addition to the planned weekly phone calls and texts, by the staff at partner agencies.

‘Getting started’ and ‘Preparation for recovery’ drug treatment groups

- Changes to commissioning of the drug group service provider and low numbers recruited resulted in the drug groups merging into a single open drug treatment ‘Getting started’ group.
- Sandwiches were supplied prior to the single drug group to support attendance from the 14th session onwards.
- The number of sessions continued beyond those initially planned due to delays in PTSD screening (see below).
- Poor attendance, the participant’s unstable behaviour and the intervention running for longer than planned affected the drug group facilitators’ ability to deliver structured content, leading to the involvement of an art worker for 4 sessions.
- PTSD screening was halted from the 29th session onwards, due to the limited remaining study time to complete the intervention, meaning the drug group became closed and only included PTSD screened participants.

For peer review only - http://bmjopen.bmj.com/site/about/guidelines.xhtml

Screening for PTSD

- Delays occurred to both the organisation of the PTSD screening and PTSD stabilisation group set up due to lack of capacity and delays in NHS trauma service staff recruitment. Therefore, some participants had long gaps between recruitment and PTSD screening and subsequent stabilisation group sessions.

PTSD 'Stabilisation' group

- Incentives of £10 vouchers per session were offered for attendance (mandatory sessions).

One-to-one EMDR therapy for PTSD

- Some sessions were scheduled in a private rented room (due to availability issues) in a local community centre and not at the SSW charity premises.
- Two participants were offered weekly one-to-one extended stabilisation sessions (8 maximum) with the clinical psychologist rather than EMDR therapy as this was deemed the most appropriate treatment.

Data collection methods

Sample size and quantitative data collection

A formal sample size calculation was not conducted as the aim was to assess feasibility.[24] Self-reported levels of illicit drug use, involvement in SSW, completion of PTSD Checklist PCL5[25] and demographics were collected at the time of consent. Attendance registers were taken at the start of each group or one-to-one session by the facilitator(s).

Qualitative data collection

With participants' verbal consent one non-participant observation of a drug group was conducted to understand delivery, examine interactions and intervention experiences, with brief notes taken during the group.[26]

In-depth semi-structured interviews were conducted with participants and service providers either face-to-face or by telephone. Participants were interviewed after intervention completion or study drop out. Consent to contact participants regarding interviews was sought at recruitment. Additional

For peer review only - http://bmjopen.bmj.com/site/about/guidelines.xhtml

written or audio recorded verbal, informed consent was obtained prior to all interviews. Interviews explored views and experiences of the intervention and how to improve acceptability. Participants received a £20 high street shopping voucher for taking part. Most service provider interviews were conducted at the end of the intervention period and also sought to understand operational issues, inter-agency working and intervention delivery.

Economic data collection

Resource use information (2018 £GBP) was collected prospectively by the agencies. Data collection focussed on four categories: Staff time, Facilities, Travel (provider funded staff and patient transport), and Materials.

Data analysis

Statistical analysis

Descriptive statistics were used to monitor recruitment and retention via CONSORT statement[27] style flowcharts, and to examine participant demographics, questionnaire responses and attendance.

Qualitative analysis

Interviews were conducted by JMK and NJ, and the audio files transcribed, anonymised and checked for accuracy. QSR NVivo 10 software was used to perform inductive thematic analysis[28] using constant comparison techniques.[29 30] A preliminary coding framework was developed by JMK and discussed with the multidisciplinary research team JH and NJ to ensure credibility and confirmability.

Cost analysis

Costs to the service provider were examined and summarised separately for each of the four intervention components. Staff costs were calculated using salaries and on-costs or generated using standard unit cost data available for health and social care professionals.[31] Facility costs were calculated based on similar space rental options. Total cost, total cost per eligible participant, total cost per session held, and total cost per session per eligible participant were calculated for each intervention component. Cost data were tabulated using principles of heat-map methodology, where colour lightness is used to communicate the magnitude of different costs.[32 33]

For peer review only - http://bmjopen.bmj.com/site/about/guidelines.xhtml

For peer review only

For peer review only - http://bmjopen.bmj.com/site/about/guidelines.xhtml

Results

Recruitment was from November 2017 to March 2018 with the intervention delivered until December 2018.

(i) Recruitment and attendance

Recruitment Process

Approximately 400 flyers and nine posters were distributed. Potential participants were spoken to by three researchers at 37 three-hour 'drop-in' sessions at the SSW charity. Of 125 women approached 84 declined screening. Reasons for decline included being too busy (n=13), not interested (n=11) or reporting not currently street sex working (n=10). Fourteen approaches were reported as repeat approaches, with seven reporting previous recruitment and four previous screening (as the researchers recruited on different weekdays and screening data was anonymous). Figure 1 details the flow of screened and recruited participants through the intervention.

Of 41 women screened, 11 were eligible and consented to participate, three were eligible but unable to give consent (two were too distressed and one had health issues preventing participation). Of the 27 ineligible women, two did not fully complete the screening questions. The main reasons for exclusion related to ineligible frequency of drug use and/or sex work. Table 1 shows the range of days since responders last street sex worked or had taken heroin/crack by those recruited and not recruited.

Of 11 participants consenting to be invited for qualitative interviews, five were uncontactable. Seven interviews were conducted with six participants (six face-to-face and one via telephone); four interviewed participants received all components of the intervention. Ten service provider interviews were conducted with representatives from the drug treatment service (n=4), the trauma service (n=2), and the SSW charity (n=4). Table 2 details quotes from the interviews to support the main results described below.

Acceptability of recruitment

The recruitment process was described as acceptable by most participants and service providers. Face-to-face recruitment was experienced as confidential, with participants reporting receiving clear

For peer review only - http://bmjopen.bmj.com/site/about/guidelines.xhtml

explanations of the study. Recruitment over five months within the SSW charity enhanced the recruiters' perceived trustworthiness. Offering multiple opportunities to participate was acceptable. Most service providers interviewed reflected that if study inclusion criteria were broadened to include less regular drug use, participants with more lifestyle stability could have been recruited, and whom may have found the intervention easier to engage with. However, some felt that creating groups with diverse levels of drug use could have negative consequences for example, risk of relapse for those who had reduced their drug use.

Group attendance

All 11 consented participants were invited to attend drug treatment groups. However, participants attendance varied throughout the study, with participants sometimes arriving late or leaving early (Table 3). Four attended 30-76% of sessions compared to 7 attenders who attended only 0-18% of sessions. The five most frequent attenders were invited to PTSD screening of which four attended and were all found to have symptoms of PTSD. All four PTSD screened participants attended 20-100% of the stabilisation groups with the clinical psychologist. At the end of the stabilisation groups, two participants were deemed suitable for EMDR therapy by the clinical psychologist and two were offered extended stabilisation sessions. All four participants missed at least two consecutive trauma treatment appointments with the clinical psychologist and were deemed to have withdrawn from the sessions. Following which, all participants were referred to further mental health services and one participant was also referred to mainstream drug services.

Facilitators to attendance

Service providers across all partner agencies sent reminders to participants, which were described as helpful and appreciated by participants. One SSW charity service provider played a vital role in encouraging attendance through reminding participants to attend, arranging transport (taxi, bus or driving participants to sessions) and also helping participants mentally prepare for the intervention. Provision of sandwich lunches before the groups was seen by service providers and participants as helpful for encouraging attendance, facilitated a relaxed start to groups and supported concentration. Vouchers were also viewed as encouraging attendance by participants and service providers.

Barriers to attendance

The unsettled nature of participant's lives was perceived as an attendance barrier and was underpinned by problematic drug use, poor adherence to opioid substitution therapy, sex work,

For peer review only - http://bmjopen.bmj.com/site/about/guidelines.xhtml

tiredness due to working nights and poor mental health. Arguments between participants, a lack of readiness to confront issues with drugs and trauma and an absence of social support were described as making attendance difficult. Delays to screening referral and trauma treatment were also reported to negatively affect participants' motivation.

(ii) Experience and acceptability of the intervention

Initial impressions

All participants perceived the intervention as valuable and welcome. Common reasons given by participants for taking part included the opportunity for 'change', greater stability and valuing the opportunity to combine mental health and drug treatment. Service providers viewed the intervention as a novel opportunity for 1) SSWs to receive mental health treatment while continuing to use drugs and 2) to address the barriers to mainstream drug treatment. Some service providers highlighted the challenge for participants to process trauma while continuing to use drugs and potential risks of sex working when receiving trauma treatment.

Service providers' views on the intervention

The drug group facilitators described enjoying delivering the groups and building good relationships with participants. However, they described some drug sessions as intense and difficult to manage due to participants' distress, accounts of trauma and chaotic behaviour. The need for appropriate support and supervision of facilitators was highlighted as a requirement to manage these challenges.

A clinical psychologist suggested that without the 're-traumatising' effects of street sex work, the effectiveness of the trauma processing in the trauma treatment might be enhanced. Service providers also proposed extending the stabilisation work to develop the effectiveness of the trauma treatment and recommended the intervention offer alternatives to EMDR to suit individual participants' needs.

Service providers said working in partnership with other specialist services to deliver the intervention was valuable, there was mutual respect and good communication between staff. It was suggested it would have been useful to have collaborative, regular case-review meetings between the services to assess the progress and needs of the participants and enhance the communication channels.

For peer review only - http://bmjopen.bmj.com/site/about/guidelines.xhtml

Participants views on the intervention

Participants described generally positive intervention experiences. They described forming meaningful relationships with the drug group facilitators and clinical psychologist. They liked that the groups were female and sex worker-only, they knew other participants already and could speak openly about, and relate to, one another's experience of trauma, drug use and street sex work. Participants also valued that the intervention was delivered at SSW charity premises, which was liked for its familiarity, safety, comfort, convenience and freedom from judgement and shame. The day of the week, time and frequency of sessions, drug group session length and group size were also acceptable to most participants and service providers. These factors overcame some of the barriers participants highlighted to attending mainstream drug services.

Impacts of the intervention

Through the intervention, participants reflected on their need to address their trauma and drug use. Some acknowledged that they were not ready to address their trauma but aspired to this in future, having had positive experiences of therapy during the intervention. Participants attributed improved wellbeing, coping strategies and perceptions of self-worth to the intervention. One participant was seen less on the SSW outreach van (an indicator of sex working) and, significantly, stopped using her working name, signifying 'taking back ownership of who she is'. One drug group facilitator felt the participants unstable lives and level of trauma would prevent them from complying with the rules of conduct in mainstream drug services and valued the flexibility of the study intervention to address these needs. They also felt that an additional positive feature of the intervention was participants being able to discuss their sex work, due to the female SSW only membership of the groups.

Participation in the intervention was described by SSW charity service providers and two participants as supporting and empowering the participants to engage with clinical and support services to address their needs. Another participant felt she used the SSW charity services less now because she needed less support.

(iii) Cost analysis

The total cost of the intervention was £11,710, with staff costs being the largest component (Table 4). The most expensive component of the intervention was the 'Getting started' sessions (which totalled £6,842). Despite having the second to lowest sub-total cost across the intervention (£1,014), the

For peer review only - http://bmjopen.bmj.com/site/about/guidelines.xhtml

stabilisation groups had the highest cost per session held (£203). Although the one-to-one sessions had the lowest cost per session held (£103), the larger number of sessions at this point resulted in this section having the highest cost per eligible participant (n=4, £724). Trauma screening had the lowest cost per eligible participant (n=5, £191)

Fidelity

The intervention was broadly delivered as intended incorporating suggested planned changes to the protocol, it was more flexible and less linear than originally planned.[24] Delays in PTSD screening meant that there was only a single drug group, which continued for longer than originally planned.

Discussion

Summary of findings

This study used a mixed methods approach to investigate the feasibility and acceptability of a novel, complex intervention to reduce illicit drug use in female drug-dependent SSWs. We demonstrated that drug-dependent SSWs could maintain attendance at female SSW-only drug group sessions and the integrated trauma-focussed treatment approach, in a trusted and supportive environment, with intensive support from SSW charity staff. Recruitment was lower than anticipated, with four participants PTSD screened and whom met criteria for PTSD. They progressed through all stages of the intervention and All four participants were ultimately able to access mental health services and one began the process of accessing mainstream drug services. Participants and service providers mostly experienced the recruitment process, the intervention and delivery mechanisms (especially the SSW-only environment) positively. Attendance and adherence barriers primarily related to the issues the intervention sought to address, namely problematic drug use, sex work, and poor mental health, rather than the acceptability of the intervention itself. The total cost of the intervention was £11,710, with staff costs dominating.

Strengths and limitations of this study

Strengths of our study were that recruiting researchers also conducted the interviews, which may have facilitated a rapport with participants and supported more open and honest reflections. Secondly, all aspects of the intervention were delivered by specialists which was necessary for this high-risk group with multiple complex co-morbidities. Thirdly, PPI formed an important part of the study and informed each stage. Fourth, recruitment over several months with multiple approaches

For peer review only - http://bmjopen.bmj.com/site/about/guidelines.xhtml

allowed SSWs many opportunities to take part. This approach took account of the rapidly changing lives of SSWs resulting in changing eligibility status as well as allowing time for them to become familiar with the researchers. Finally, recruitment within a trusted agency may have had a positive influence on recruitment.

This feasibility study provides only preliminary information on the intervention performance and cost and does not examine the effectiveness or potential for reducing costs in other parts of the health service or wider society. Delays due to changes to service provision are likely to have adversely influenced study recruitment and retention, with delays resulting in higher service costs, but reflect the real-life issues facing multi-agency work.

Comparison with other research

This feasibility study is the first, to our knowledge, to address previously highlighted barriers to effective drug treatment for SSWs.[15 22 23] Through incorporating female SSW-only drug groups alongside an intervention with specialised trauma treatment, delivered in a female SSW-only setting by female staff[22 24] we showed how an integrated treatment approach in this complex vulnerable group can be feasibly implemented and delivered.

This study is the first interventional study to address trauma symptoms as part of the drug treatment process, identified as important for this group who have been found to have high levels of poor mental health,[16 18]particularly trauma,[5 16 17] which has been highlighted as contributing to poor drug treatment outcomes.[7 8] The intervention took account of SSWs frequent experience of abuse and violence,[34 35] and recommendations for female-only trauma-focussed drug treatment interventions[22 36 37] for treatment of PTSD and long term drug use reduction. Some of the characteristics the intervention sought to address presented as barriers to attendance and retention, however these are common in studies trying to effect behaviour changes within vulnerable groups.[23 38]

Previous SSW-focussed interventions aiming to reduce drug use[10-13 14] were unable to demonstrate strong evidence of a positive effect[15] suggesting the need for a novel approach with evidence of efficacy assessed through a robust methodological approach. The highlighted barriers to attendance, engagement and delivery of the intervention are in keeping with other studies[15 23 38] but indicate that further changes to the DUSSK feasibility study design are likely to be required in future studies.

For peer review only - http://bmjopen.bmj.com/site/about/guidelines.xhtml

Conclusion and implications for service provision and research

The inclusion criteria were informed by PPI, clinical expertise and academic literature, and targeted women with chaotic lives who would likely benefit the most from a trauma-based intervention. Only women whom sold sex on the street *and* used heroin or crack cocaine, at least once a week, were eligible to take part. Many women were sex working and using drugs but less frequently than previous evidence indicated, and so were excluded. Both participants and service providers reported that to enable greater recruitment and intervention engagement, the eligibility criteria could better target those with more stability in their lives and hence fewer barriers to participation. However, due to the chaotic nature of participants' lives, recruitment and retention were at times challenging, highlighting a trade-off between engaging women whom might benefit the most from trauma treatment versus recruiting more stable women. Future research in this area should consider this trade-off carefully, allowing flexibility within the research process to ensure the inclusion criteria are appropriate and realistic.

All four of the screened participants met criteria for PTSD, revealing the unmet need for treatment and the severity of trauma in SSWs' lives. Although small in numbers, meeting PTSD criteria was unsurprising as it agrees with previous research.[22 36 37] Further data to understand the extent of PTSD symptoms in this group is therefore recommended. Overall, the experiences described by those receiving the intervention suggest it is an acceptable approach to reducing SSWs drug use. The three services were able to work together effectively despite setbacks such as changing contracts and service pressures.

In order to support future interventional trials in this important field where there are relatively few effectiveness studies we would recommend the following study refinements for consideration.

1. The intervention could include women with more stability in their lives to increase recruitment and retention.
2. Regular meetings throughout the study enabling all service providers involved in intervention delivery to express concerns and seek to understand participants needs from the perspective of different professionals so there is effective multi-agency support for individual participants where needed.
3. Support and encouragement for participant engagement through provision of transport to and refreshments prior to treatment sessions.
4. Intervention flexibility and responsiveness in offering trauma-focussed alternatives to EMDR which may be more suitable for individual participants needs.

For peer review only - http://bmjopen.bmj.com/site/about/guidelines.xhtml

5. An extended trauma therapy programme to accommodate the complexity of SSW needs.

For peer review only

For peer review only - http://bmjopen.bmj.com/site/about/guidelines.xhtml

Additional information

Acknowledgements

The authors are extremely grateful to all the women who have participated in the study; all service providers and all other staff whose participation made this study possible. They would like to thank the PPI group for their time, thoughts and suggestions. We are grateful to the following organisations and individuals who have helped with the study for their time, expertise and support: Developing Health and Independence, Bristol Drugs Project, Avon & Wiltshire Mental Health Partnership NHS Trust and One25 charity, Katie Warner, Lucy Pettler, Rosie Davies, Maggie Telfer, Elinor Griffiths, Gill Nowland, Sophie Ramsden, Elaine Driver, Anna Smith, Rhea Warner, Jennifer Riley, Tracey Tudor, Madeline Saunders, Charlotte Hignell, Sophie Banks, Jane Bowman, Sarah Shatwell, Katrina Turner, Hasina Downie, Jo Daniels and Louisa Chowen.

Author Contributions

NJ, NMR, JH, JMK, RP, DW, JM, JMa, MJL and JC are responsible for the study design, collection of data and analysis. NMR, RP, NJ and JH are responsible for study management and coordination. JMK and NJ conducted the interviews. JMK led the qualitative analysis in collaboration with NJ and JH. RP conducted the quantitative analysis. MJL, RP and JC conducted the costing study. NMR, RP, JMK, MJL, JC, NJ and JH drafted the paper. All authors read, commented on and approved the final manuscript.

Funding

The research is supported by a National Institute for Health Research (NIHR) Clinic Trials Fellowship awarded to NJ, the National Institute for Health Research Collaboration for Leadership in Applied Health Research and Care West (NIHR CLAHRC West), now recommissioned as NIHR Applied Research Collaboration West (NIHR ARC West) and Research Capability Funding awarded by University Hospitals Bristol NHS Foundation Trust. JMK is partly funded by NIHR Health Protection Research Unit in Evaluation of Interventions. The views expressed are those of the authors and not necessarily those of the NIHR or the Department of Health and Social Care.

Competing interests

None declared

Data availability statement

For peer review only - http://bmjopen.bmj.com/site/about/guidelines.xhtml

All data relevant to the study are included in the article.

For peer review only

For peer review only - http://bmjopen.bmj.com/site/about/guidelines.xhtml

References

1. Cusick L, Martin A, May T. Vulnerability and involvement in drug use and sex work. Home Office research study 268. London: Home Office, 2003.
2. Deering KN, Kerr T, Tyndall MW, et al. A peer-led mobile outreach program and increased utilization of detoxification and residential drug treatment among female sex workers who use drugs in a Canadian setting *Drug & Alcohol Dependence* 2011;113(1):46-54.
3. Morris D, Lemus H, Wagner K D, et al. Factors associated with pathways toward concurrent sex work and injection drug use among female sex workers who inject drugs in northern Mexico. *Addiction* 2013;108:161-70.
4. Jeal N, Salisbury C, Turner K. The multiplicity and interdependency of factors influencing the health of street-based sex workers: a qualitative study. *Sex Transm Infect* 2008;84(5):381-5. doi: 10.1136/sti.2008.030841 [published Online First: 2008/07/04]
5. Gorry J, Roen K, Reilly J. Selling your self? The psychological impact of street sex work and factors affecting support seeking. *Health Soc Care Community* 2010;18(5):492-9. doi: 10.1111/j.1365-2524.2010.00925.x [published Online First: 2010/06/30]
6. Jeal N, Salisbury C. Health needs and service use of parlour-based prostitutes compared with street-based prostitutes: a cross sectional survey. *BJOG* 2007;114:875-81.
7. Burnette M LE, Ilgen M, Frayne S, Mayo J, Weitlauf J. Prevalence and health correlates of prostitution among patients entering treatment for substance use disorders. *Archives of General Psychiatry* 2008;65(3):337 - 44.
8. Burnette M SR, Timko C, Ilgen M. Impact of substance-use disorder treatment on women involved in prostitution: substance use, mental health and prostitution one year after treatment. *Journal of Studies on Alcohol and Drugs* 2009;70:32-40.
9. Jeal N, Macleod J, Salisbury C, et al. Identifying possible reasons why female street sex workers have poor drug treatment outcomes: a qualitative study. *BMJ Open* 2017;7(3):e013018. doi: 10.1136/bmjopen-2016-013018 [published Online First: 2017/03/25]
10. Sherman SG, German D, Cheng Y, et al. The evaluation of the JEWEL project: An innovative economic enhancement and HIV prevention intervention study targeting drug using women involved in prostitution. *Aids Care-Psychological and Socio-Medical Aspects of Aids/Hiv* 2006;18(1):1-11. doi: 10.1080/09540120500101625
11. Surratt HL, Inciardi JA. An effective HIV risk-reduction protocol for drug-using female sex workers. *J Prev Interv Community* 2010;38(2):118-31. doi: 10.1080/10852351003640732 [published Online First: 2010/04/15]
12. Gunne LM, Gronbladh L, Petersson S. [Methadone treatment in the prevention of AIDS. Heroin-dependent prostitutes are an important target]. *Lakartidningen* 1986;83(49):4194-6. [published Online First: 1986/12/03]
13. Litchfield J, Maronge A, Rigg T, et al. Can a targeted GP-led clinic improve outcomes for street sex workers who use heroin? *The British journal of general practice : the journal of the Royal College of General Practitioners* 2010;60(576):514-6. doi: 10.3399/bjgp10X514774 [published Online First: 2010/07/03]
14. Yahne CE, Miller WR, Irvin-Vitela L, et al. Magdalena Pilot Project: motivational outreach to substance abusing women street sex workers. *J Subst Abuse Treat* 2002;23(1):49-53. [published Online First: 2002/07/20]
15. Jeal N, Macleod J, Turner K, et al. Systematic review of interventions to reduce illicit drug use in female drug-dependent street sex workers. *BMJ Open* 2015;5(11):e009238. doi: 10.1136/bmjopen-2015-009238 [published Online First: 2015/11/20]
16. El-Bassel N, Schilling R, Irwin K, et al. Sex trading and psychological distress among women recruited from the streets of Harlem. *American Journal Public Health* 1997;87:66-70.
17. Chukakov B, Ilan K, Belmaker RH, et al. The motivation and mental health of sex workers. *Journal of Sex & Marital Therapy* 2002;.28(4):pp. doi: 10.1080/00926230290001439

For peer review only - http://bmjopen.bmj.com/site/about/guidelines.xhtml

18. Roxburgh A, Degenhardt L, J C, et al. Drug Dependence and Associated Risks Among Female Street-Based Sex Workers in the Greater Sydney Area, Australia. *Substance Use & Misuse* 2008;43(8-9):1202-17.
19. Mellor R, Lovell A. The lived experience of UK street-based sex workers and the health consequences: an exploratory study. *Health promotion international* 2012;27(3):311-22. doi: 10.1093/heapro/dar040 [published Online First: 2011/07/06]
20. Reynolds M, Mezey G, Chapman M, et al. Co-morbid post-traumatic stress disorder in a substance misusing clinical population. *Drug and alcohol dependence* 2005;77(3):251-58. doi: https://doi.org/10.1016/j.drugalcdep.2004.08.017
21. Cloitre M, Courtois CA, Charuvastra A, et al. Treatment of complex PTSD: Results of the ISTSS expert clinician survey on best practices. *Journal of Traumatic Stress* 2011;24(6):615-27. doi: 10.1002/jts.20697
22. Roberts NP, Roberts PA, Jones N, et al. Psychological therapies for post-traumatic stress disorder and comorbid substance use disorder. *Cochrane Database Syst Rev* 2016;4(4):CD010204. doi: 10.1002/14651858.CD010204.pub2 [published Online First: 2016/04/05]
23. Bailey K, Trevillion K, Gilchrist G. What works for whom and why: A narrative systematic review of interventions for reducing post-traumatic stress disorder and problematic substance use among women with experiences of interpersonal violence. *Journal of Substance Abuse Treatment* 2019;99:88-103. doi: 10.1016/j.jsat.2018.12.007
24. Jeal N, Patel R, Redmond NM, et al. Drug use in street sex workers (DUSSK) study protocol: a feasibility and acceptability study of a complex intervention to reduce illicit drug use in drug-dependent female street sex workers. *BMJ Open* 2018;8(11):e022728. doi: 10.1136/bmjopen-2018-022728 [published Online First: 2018/11/06]
25. Bovin MJ, Marx BP, Weathers FW, et al. Psychometric properties of the PTSD Checklist for Diagnostic and Statistical Manual of Mental Disorders-Fifth Edition (PCL-5) in veterans. *Psychol Assess* 2016;28(11):1379-91. doi: 10.1037/pas0000254 [published Online First: 2016/11/04]
26. Emerson RM, Fretz RI, Shaw LL, et al. Writing ethnographic fieldnotes. Chicago and London: University of Chicago Press 1995.
27. Schulz KF, Altman DG, Moher D, et al. CONSORT 2010 statement: updated guidelines for reporting parallel group randomised trials. *BMJ* 2010;340:c332. doi: 10.1136/bmj.c332 [published Online First: 2010/03/25]
28. Braun V, Clarke V. Using thematic analysis in psychology. *Qualitative Research in Psychology* 2006;3(2):77-101.
29. Glaser B, Strauss A. The Constant Comparative method of Qualitative Analysis. In the Discovery of Grounded Theory: Strategies for Qualitative Research Chicago: Aldine 1967:101-158.
30. Charmaz K. Constructing grounded theory: a practical guide through qualitative analysis. London: Sage Publications, Inc 2006.
31. Curtis LB, A. . Unit Costs of Health and Social Care University of Kent, Canterbury: Personal Social Services Research Unit 2018.
32. Kirk A. Data Visualization: a successful design process: Packt Publishing Ltd 2012.
33. Iliinsky N, Steele J. Designing data visualizations: Representing informational Relationships: O'Reilly Media, Inc. 2011.
34. Ulibarri MD, Hiller SP, Lozada R, et al. Prevalence and characteristics of abuse experiences and depression symptoms among injection drug-using female sex workers in Mexico. *J Environ Public Health* 2013;2013:631479. doi: 10.1155/2013/631479 [published Online First: 2013/06/06]
35. Monica D. Ulibarri, Steffanie A. Strathdee, Remedios Lozada, et al. Prevalence and Correlates of Client-Perpetrated Abuse Among Female Sex Workers in Two Mexico–U.S. Border Cities. *Violence Against Women* 2014;20(4):427– 45.

For peer review only - http://bmjopen.bmj.com/site/about/guidelines.xhtml

36. Greenfield SF, Brooks AJ, Gordon SM, et al. Substance abuse treatment entry, retention, and outcome in women: a review of the literature. *Drug and alcohol dependence* 2007;86(1):1-21. doi: 10.1016/j.drugalcdep.2006.05.012 [published Online First: 2006/06/09]
37. Wiechelt SA, Shdaimah CS. Trauma and Substance Abuse Among Women in Prostitution: Implications for a Specialized Diversion Program. *Journal of Forensic Social Work* 2011;1(2):159-84. doi: 10.1080/1936928x.2011.598843
38. Bailey K, Trevillion K, Gilchrist G. "We have to put the fire out first before we start rebuilding the house": practitioners' experiences of supporting women with histories of substance use, interpersonal abuse and symptoms of post-traumatic stress disorder. *Addiction Research & Theory* 2019:1-9. doi: https://doi.org/10.1080/16066359.2019.1644323

For peer review only

Figure 1 Flow of participants through the DUSSK study

For peer review only

For peer review only - http://bmjopen.bmj.com/site/about/guidelines.xhtml

Table 1 Characteristics of screened women

	Screened not recruited (N=30)		Screened and recruited (N=11)	
	**N (%) ***	**Median (range)**	**N (%) ***	**Median (range)**
Female	27 (90)		11 (100)	
Age	23 (77)	37 (26-55)	11 (100)	38 (23-53)
Ever sold sex on the street?				
Yes	26 (87)		11 (100)	
No	1 (3)		-	
How many days since last worked on the street?	23 (77)	60 (1-2920)	11 (100)	7 (1-28)
How often usually sell sex on street?				
Daily	6 (20)		3 (27)	
Weekly	5 (17)		8 (73)	
Less than weekly	16 (53)		-	
Ever used street drugs				
Yes	26 (87)		11 (100)	
No	1 (3)		-	
Ever used heroin	23 (77)		9 (82)	
Days since last used heroin	19 (63)	2 (0-731)	9 (82)	1 (0-6)
Ever used crack cocaine	23 (77)		11(100)	
Days since last used crack cocaine	21 (70)	2 (0-2922)		1 (0-4)
How often use heroin and/or crack cocaine?				
Daily	11 (37)		7 (64)	
Weekly	4 (13)		4 (36)	
Less than weekly	9 (30)		-	
Has an opioid substitute script	13 (43)		6 (55)	
Script type				
Buprenorphine/Subutex®	-		1 (9)	
Methadone	13 (43)		5 (45)	
Used other drugs:				
Alcohol	3 (10)		1 (9)	
Amphetamine	1 (3)		-	
Cannabis	5 (17)		5 (45)	
Spice	2 (7)		-	
MDMA (Ecstasy)	1 (3)			
Tramadol	1 (3)		-	
Sleeping tablets	-		1 (9)	

*N and % of those that provided data

For peer review only

Table 2 Qualitative quotes

Theme/Sub-theme	Quotes
**Recruitment and retention**	
**Acceptability**	*[Face-to-face recruitment] worked well, it wasn't intrusive, you weren't pushy, you know you blended in within the drop-in setting. So I think the women felt that if they did wanna buy into it they would, there was no pressure there. So I think that was done really sensitively.* Service provider 6<br><br>*It [recruitment] was very sort of like confidential and actually it was quite nice 'cause, yeah no one really knew what I was doing when I was doing summut, you know what I mean, which is – like it don't usually happen like that. Everyone knows what I'm doing all the time.* Participant 7
**Improvements**	*I think from a clinical point of view if you remove that criteria [sex work at least once a week in the last calendar month] and then of course there's more chance of getting people through to the finish line to be able to be ready for treatment at the end.* Service provider 4<br><br>*Actively drug using? Yes, that makes sense (…). If they've been able to bring that down themselves maybe another service would be better. Like, what this offered, it's specialistic in this. So if you was able to manage to a level yourself, maybe you don't need [the intervention]… I'm not sure, I think that would be an interesting conversation because if they could bring it down themselves, they'd probably be a lot more stable and a lot more reliable to actually get to the EMDR .* Service provider 7<br><br>*So I think if you were to extend the period of time and say 'Oh actually do you know if you've used within the last three months you can participate in the study and then someone who's three months abstinent or reducing from their street heroin use or their crack use is then exposed to somebody who's going no no no man I'm using up like a party every night'. There'd be that ethical thing within it but it would be nice to see the study opened up to a wider cohort.* Service provider 2
**Facilitators to attendance**	
Encouragement and support to attend	*I would say that I've been quite integral in regards to developing relationships with the women, contacting them for both their individual one to ones and stabilisation groups and also their Thursday DUSSK groups as well. So just keeping that contact going if they were coming in, in our drop-in service I would see them and then sort of give them reminders, did they want little welfare calls, that type of thing.* Service provider 6

Transport	*It was more of a focus thing where you know she [service provider 6] sort of like coached us as we went down, like you know keeping us like sort of aware of what we've got to be thinking of doing and making sure that, you know, there's nothing wrong.* Participant 7
Food provision	*I was turning up and I was like sort of god like hanging out for (…) that lunch. It was like, not the reason I was turning up but the main reason why I could (…). There is light at the end of the tunnel, you know you're gonna be nourished and fed.' You're gonna be able to concentrate as well.* Participant 7
**Barriers to attendance**	
Unstable lifestyles	*My mental problems, my drug use, everything, just my life, it gets in the way [of attendance].* Participant 1
Mental health	*They're so low resourced, they just don't have the distress tolerance to be able to cope with any more distress, they're already facing so much. Even things like their housing and threats of eviction.* Service provider 8<br><br>*My home life was getting a bit chaotic. My depression was getting really bad as well. So, yeah, and I was waiting for my antidepressants to work but they took a while. Yeah, it was just my depression, that's all.* My anxiety. Participant 6
Sex work	*If I've been working the night before there's no way I could have attended because I'm too tired, because you work all night.* Participant 4
Delays between treatment stages	*It took a little bit of a while and also for them to access their stabilisation groups then their one to ones. I think we may have lost some of the interest.* Service provider 6
**Experience and acceptability of the intervention**	
Initial impressions	*There aren't many services out there, which will offer individual, tailorised counselling and support to the women who have got dual diagnosis and you know mental health, drug misuse. So this study was unique and I think that's what we were all so passionate and so behind it because it was giving the women an opportunity.* Service provider 6
Reason for participating	*I just felt so alone and afraid and stuck and just needed to see if there was some way that I might be able to gain something so – really, if I'm willing to put myself out on the street and sell myself to a complete stranger, knowing that I might die, whatever, so it kind of … I felt I needed to understand why I needed to do this... So it's about me owning my power, and about not letting myself feel as shit about myself as I have done*. Participant 5

For peer review only - http://bmjopen.bmj.com/site/about/guidelines.xhtml

	The post-traumatic stress [treatment] is – is a way of like sort of detoxing your brain. So, you know finding a reason why you do these drugs (…) to like sort of be the reason for me to like say 'Well, I've got to stop now.' You know and get off it. Participant 7
Service providers views on the intervention	*I guess that people thought they weren't going to talk about their traumas [in the drug groups] but if somebody's been raped last night, they're going to need to talk about it, so we were here dealing with that stuff on the spot and then we didn't have no-one to go away and talk about it.* Service provider 10<br><br>*It's very hard to do trauma processing when women to some degree are being traumatised and then having to self-medicate against all of that and then you're trying to work on quite deep attachment developmental trauma stuff from a long time ago. (…) I'd say that trauma processing would be more successful with women who have maybe made a very strong commitment to stop [sex] working.* Service provider 8<br><br>*I would offer it [EMDR] as part of a – as a range of things that are offered…we'd say 'You can have EMDR, trauma focus CBT [Cognitive Behavioural Therapy] or narrative exposure therapy and you'd kind of match the person to what you thought they might be more suited to*. Service provider 8<br><br>*I think they had huge admiration for the workers at [SSW charity], and found them friendly and supportive, but…there wasn't a specific, I don't know, once a month structured 'let's talk about the women and how they've been in the month.* Service provider 9
Participants views of the intervention	*I enjoyed going down there. (...) We had a good laugh and learned something while we were doing it.* Participant 6<br><br>*With it being all woman and not mixed going to (mainstream drug treatment service provider) and doing groups where men are involved is like, I didn't really want to do it but here because it's all women and I know most of the women that come here, we've all been through it, hence why we all come here. So one way or another we've all been through something that we can all relate to.* Participant 3
Intervention characteristics	*It's [SSW charity] familiar and it's comfortable and it's safe.* Service provider 5

For peer review only - http://bmjopen.bmj.com/site/about/guidelines.xhtml

	The groups weren't too big, so you sort of – I knew the people that were coming to the groups which was better, so we'd sort of you know built up a rapport. Participant 4
Impacts of the intervention	*I'm just going to stop [drug use], I'm ready and I'm kind of already preparing for that, so it's kind of brought me to a close, and I mean that as well. Personally it's like, I'm ready, bring it on, I'm like do you know what I've been raped, I've been beaten I've stuck needles in myself(…) I'm done, I'm not playing this game anymore, I deserve better.* Participant 5<br><br>*It's just made (…) me realise I'm not just a, like, drug addict, sex worker. I'm a real person and I've got feelings and, you know, I've got potential. You know, yeah, they [service providers] build me up a lot.* Participant 6<br><br>*When she* [participant 6] *started with [name of intervention] study and she was coming to her Thursday [drug] groups, she didn't want to be associated with street sex working. So she said 'Can you call me (own name rather than working name)?' I could have cried (…). She was owning her own name and taking back ownership of who she is rather than somebody who was street sex working.* Service provider 6<br><br>*Their chaoticness. (…) To manage that in a [mainstream drug service] group setting would be difficult and I'm not sure how they would manage that. I just know how much regularly how they've turned up [to the intervention drug treatment groups] chaotic and they've turned up leaking out trauma. … I'm far from confident that they would be able to sit under them [mainstream drug service] rules enough to be a part of what it is for here[research study], due to the level of flexibility here and that they would be able to talk about what their problem is without mentioning what they do and that might make them vulnerable* Service provider 7
Fidelity	*I guess we were kind of thinking of it in a really linear sense, that the women would engage in the drug groups and then reduce their drug use to then move on to the next group and I'm not sure that that actually happened in reality.*<br>Service provider 5<br><br>*In the beginning we went in doing the same sort of work that we would do here [mainstream drug services], and it's getting them to look at their behaviour, and the consequences of it and stuff, and it didn't work with these women, it's too much, too direct.* Service provider 10

For peer review only - http://bmjopen.bmj.com/site/about/guidelines.xhtml

Table 3 Attendance and retention of participants

	Open group Getting started			Attended Trauma Screening	Closed group Getting started			5 mandatory Stabilisation group sessions		One-to-one sessions				Referred to which services:
Participant	Eligible sessions	Attended sessions*	% Attended		Eligible sessions	Attended sessions	% Attended	Attended sessions	% Attended	Treatment	Eligible sessions**	Attended sessions*	% Attended	
1	28	10	36	Yes	26	4	15	3	60	Ext. stabilisation	8[3]	1 (1)	13	Mental health
2	28	5	18	No										
3	28	1 (1)	4	NA										
4	27	8	30	Yes	26	11	42	5	100	EMDR	12[3]	4 (1) $	33	Mental health
5	25	3	12	NA										
6	25	19 (5)	76	Yes	26	15	58	4	80	EMDR	12[2]	8 (4) $	67	Mental health & mainstream drug
7	23	0	0	NA										
8	22	0	0	NA										
9	20	7 (2)	35	Yes	26	3	12	1	20	Ext. stabilisation	8[4]	0	0	Mental health
10	19	0	0	NA										
11	19	0	0	NA										
Total		53		4		33		13				13		

*N session participant arrived late/left early indicated in superscript round brackets; **N sessions cancelled due to non-attendance in square brackets; NA=Not actively invited to sessions; $ Includes one review session; Ext. stabilisation=Extended stabilisation, EMDR=Eye movement desensitization and reprocessing therapy

For peer review only - http://bmjopen.bmj.com/site/about/guidelines.xhtml

Table 4 Health Economics

	1. Getting started		2. Trauma screening		3. Stabilisation group		4. One-to-one sessions	
**Service description**								
Session lengths - range	90 - 120 minutes [1]		60 minutes		60 minutes		60 - 90 minutes [2]	
Number of sessions held - total	52		8		5		28	
Eligible participants[3] - total	n = 11		n = 5[4]		n = 4		n = 4	
Attendees per session - range	0 - 4		1		2 - 4		1	
**Costs**	**Sub-total £**	**£ per ppt**	**Sub-total £**	**£ per ppt**	**Sub-total £**	**£ per ppt**	**Sub-total £**	**£ per ppt**
**A. Staff**								
Service manager	£1,359.76	£123.61	£95.09	£19.02	£47.54	£11.89	£266.25	£66.56
Drug group facilitators[5]	£3,000.20	£272.75	£0.00	£0.00	£0.00	£0.00	£0.00	£0.00
Art worker	£123.98	£11.27	£0.00	£0.00	£0.00	£0.00	£0.00	£0.00
Clinical Psychologist	£0.00	£0.00	£636.00	£127.20	£600.00	£150.00	£2,040.00	£510.00
**B. Facilities**								
Space rental	£1,270.50	£115.50	£224.00	£44.80	£140.00	£35.00	£574.00	£143.50
**C. Travel**								
Transporting materials	£73.78	£8.44	£0.00	£0.00	£11.90	£2.98	£0.00	£0.00
Car lifts for service-users (petrol)	£38.72	£3.52	£2.24	£0.45	£3.36	£0.84	£1.20	£0.30
Public transport for participants	£70.20	£6.38	£0.00	£0.00	£3.90	£0.98	£3.90	£0.98
Taxis for participants	£55.00	£5.00	£0.00	£0.00	£62.20	£15.55	£11.00	£2.75
**D. Materials**								
Art supplies	£30.00	£2.73	£0.00	£0.00	£0.00	£0.00	£0.00	£0.00
Stationary	£0.00	£0.00	£0.00	£0.00	£5.00	£1.25	£0.00	£0.00
Voucher incentives	£0.00	£0.00	£0.00	£0.00	£130.00	£32.50	£0.00	£0.00
Refreshments[6]	£820.00	£74.55	£0.00	£0.00	£10.00	£.2.50	£0.00	£0.00
**Summary**								
Total cost	£6,842.13	£622.01	£957.33	£191.47	£1,013.90[7]	£253.48	£2,896.35	£724.09
Total cost per session	£131.58	£11.96	£119.67	£23.93	£202.78	£50.70	£103.44	£25.86

Note: 1 = Sessions were originally were 90 minutes, however when sandwiches were provided drug group facilitators arrived 30 minutes prior to session to be with participants while they ate, 2 = One-to-one EMDR sessions were 90 minutes and one-to-one Stabilisation sessions were 60 minutes, 3 = participants, 4 = Five participants were eligible for screening, however only four participants were successfully screened, 5 = Getting started groups were facilitated by two drug group facilitators, and 6 = Sandwiches and biscuits, 7 = Total cost without vouchers would have been £883.90 .

Heat map description: **£0.00** | **£0.01 - £99.99** | **£100 - £499.99** | **£500.00 - £1999.99** | **£2000.00 +**

For peer review only - http://bmjopen.bmj.com/site/about/guidelines.xhtml

Figure legend

*Numbers may include more than one approach to potential participants by different recruiters

For peer review only

For peer review only - http://bmjopen.bmj.com/site/about/guidelines.xhtml

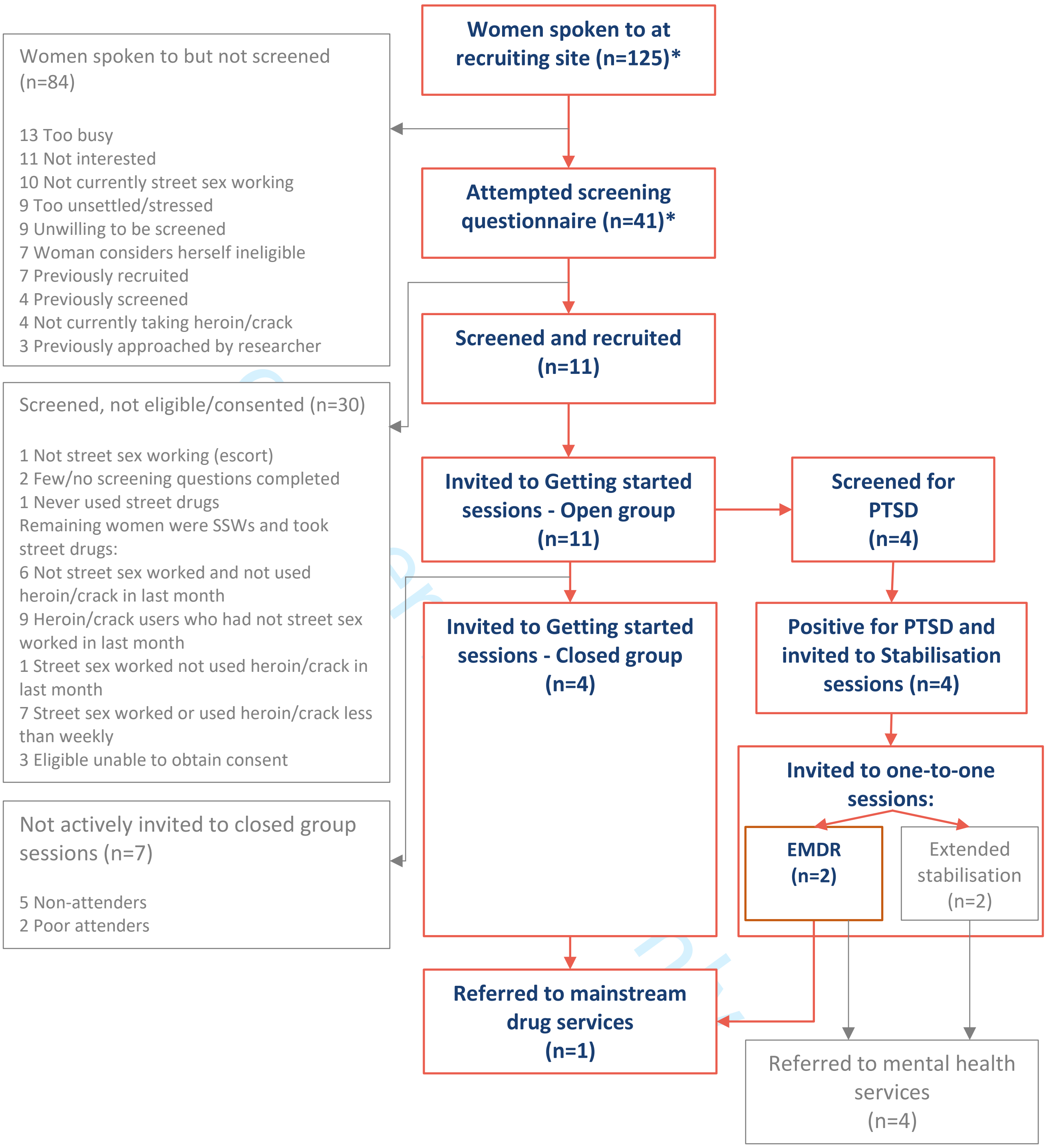

*Numbers may include more than one approach to potential participants by different recruiters

Reporting checklist for cohort study.

Based on the STROBE cohort guidelines.

Instructions to authors

Complete this checklist by entering the page numbers from your manuscript where readers will find each of the items listed below.

Your article may not currently address all the items on the checklist. Please modify your text to include the missing information. If you are certain that an item does not apply, please write "n/a" and provide a short explanation.

Upload your completed checklist as an extra file when you submit to a journal.

In your methods section, say that you used the STROBE cohortreporting guidelines, and cite them as:

von Elm E, Altman DG, Egger M, Pocock SJ, Gotzsche PC, Vandenbroucke JP. The Strengthening the Reporting of Observational Studies in Epidemiology (STROBE) Statement: guidelines for reporting observational studies.

For peer review only

		Reporting Item	Page Number
**Title and abstract**			
Title	#1a	Indicate the study’s design with a commonly used term in the title or the abstract	1
Abstract	#1b	Provide in the abstract an informative and balanced summary of what was done and what was found	2
**Introduction**			
Background / rationale	#2	Explain the scientific background and rationale for the investigation being reported	4
Objectives	#3	State specific objectives, including any prespecified hypotheses	4
**Methods**			
Study design	#4	Present key elements of study design early in the paper	5
Setting	#5	Describe the setting, locations, and relevant dates, including periods of	5

		recruitment, exposure, follow-up, and data collection	
Eligibility criteria	#6a	Give the eligibility criteria, and the sources and methods of selection of participants. Describe methods of follow-up.	5
Eligibility criteria	#6b	For matched studies, give matching criteria and number of exposed and unexposed	N/A
Variables	#7	Clearly define all outcomes, exposures, predictors, potential confounders, and effect modifiers. Give diagnostic criteria, if applicable	8
Data sources / measurement	#8	For each variable of interest give sources of data and details of methods of assessment (measurement). Describe comparability of assessment methods if there is more than one group. Give information separately for for exposed and unexposed groups if applicable.	7
Bias	#9	Describe any efforts to address potential sources of bias	N/A
Study size	#10	Explain how the study size was arrived at	7
Quantitative variables	#11	Explain how quantitative variables were handled in the analyses. If applicable, describe which groupings were chosen, and why	7
Statistical methods	#12a	Describe all statistical methods, including those used to control for confounding	8
Statistical methods	#12b	Describe any methods used to examine subgroups and interactions	N/A
Statistical methods	#12c	Explain how missing data were addressed	N/A
Statistical methods	#12d	If applicable, explain how loss to follow-up was addressed	N/A
Statistical methods	#12e	Describe any sensitivity analyses	N/A
**Results**			
Participants	#13a	Report numbers of individuals at each stage of study—eg numbers potentially eligible, examined for eligibility, confirmed eligible, included in the study, completing follow-up, and analysed. Give information separately for for exposed and unexposed groups if applicable.	20

For peer review only

Participants	#13b	Give reasons for non-participation at each stage	9
Participants	#13c	Consider use of a flow diagram	20
Descriptive data	#14a	Give characteristics of study participants (eg demographic, clinical, social) and information on exposures and potential confounders. Give information separately for exposed and unexposed groups if applicable.	9
Descriptive data	#14b	Indicate number of participants with missing data for each variable of interest	21
Descriptive data	#14c	Summarise follow-up time (eg, average and total amount)	26
Outcome data	#15	Report numbers of outcome events or summary measures over time. Give information separately for exposed and unexposed groups if applicable.	9
Main results	#16a	Give unadjusted estimates and, if applicable, confounder-adjusted estimates and their precision (eg, 95% confidence interval). Make clear which confounders were adjusted for and why they were included	9-12
Main results	#16b	Report category boundaries when continuous variables were categorized	N/A
Main results	#16c	If relevant, consider translating estimates of relative risk into absolute risk for a meaningful time period	N/A
Other analyses	#17	Report other analyses done—e.g., analyses of subgroups and interactions, and sensitivity analyses	9-12
**Discussion**			
Key results	#18	Summarise key results with reference to study objectives	13
Limitations	#19	Discuss limitations of the study, taking into account sources of potential bias or imprecision. Discuss both direction and magnitude of any potential bias.	13
Interpretation	#20	Give a cautious overall interpretation considering objectives, limitations, multiplicity of analyses, results from similar studies, and other relevant evidence.	15
Generalisability	#21	Discuss the generalisability (external validity) of the study results	14
**Other Information**			

For peer review only

Funding	#22	Give the source of funding and the role of the funders for the present study and, if applicable, for the original study on which the present article is based	16

The STROBE checklist is distributed under the terms of the Creative Commons Attribution License CC-BY. This checklist was completed on 16. December 2019 using https://www.goodreports.org/, a tool made by the EQUATOR Network in collaboration with Penelope.ai

For peer review only

BMJ Open

Drug Use in Street Sex worKers (DUSSK) study – results of a mixed methods feasibility study of a complex intervention to reduce illicit drug use in drug dependent female sex workers

Journal:	*BMJ Open*
Manuscript ID	bmjopen-2019-036491.R1
Article Type:	Original research
Date Submitted by the Author:	02-Aug-2020
Complete List of Authors:	Patel, Rita; University Hospitals Bristol NHS Foundation Trust, NIHR ARC West; University of Bristol, Population Health Sciences<br>Redmond, Niamh; University Hospitals Bristol NHS Foundation Trust, NIHR ARC West; University of Bristol, Population Health Sciences<br>Kesten, Joanna; University Hospitals Bristol NHS Foundation Trust, NIHR ARC West; University of Bristol, Population Health Sciences & NIHR Health Protection Research Unit in Evaluation of Interventions<br>Linton, Myles-Jay; University Hospitals Bristol NHS Foundation Trust, NIHR ARC West; University of Bristol, Population Health Sciences & Health Economics Bristol<br>Horwood, Jeremy; University Hospitals Bristol NHS Foundation Trust, NIHR ARC West; University of Bristol, Population Health Sciences & Centre for Academic Primary Care<br>Wilcox, David ; Avon and Wiltshire Mental Health Partnership NHS Trust, Acer Unit, Blackberry Hill Hospital, Manor Road,<br>Munafo , Jess; Avon and Wiltshire Mental Health Partnership NHS Trust, Acer Unit, Blackberry Hill Hospital, Manor Road,<br>Coast, Joanna; University Hospitals Bristol NHS Foundation Trust, NIHR ARC West; University of Bristol, Population Health Sciences & Health Economics Bristol<br>Macleod, John; University Hospitals Bristol NHS Foundation Trust, NIHR ARC West; University of Bristol, Population Health Sciences & Centre for Academic Primary Care<br>Jeal, Nicola; University Hospitals Bristol NHS Foundation Trust, NIHR ARC West; University of Bristol, Population Health Sciences
**Primary Subject Heading**:	Addiction
Secondary Subject Heading:	Health services research, Mental health, Public health, Health economics
Keywords:	QUALITATIVE RESEARCH, MENTAL HEALTH, HEALTH ECONOMICS, TRAUMA MANAGEMENT, Substance misuse < PSYCHIATRY

I, the Submitting Author has the right to grant and does grant on behalf of all authors of the Work (as defined in the below author licence), an exclusive licence and/or a non-exclusive licence for contributions from authors who are: i) UK Crown employees; ii) where BMJ has agreed a CC-BY licence shall apply, and/or iii) in accordance with the terms applicable for US Federal Government officers or employees acting as part of their official duties; on a worldwide, perpetual, irrevocable, royalty-free basis to BMJ Publishing Group Ltd ("BMJ") its licensees and where the relevant Journal is co-owned by BMJ to the co-owners of the Journal, to publish the Work in this journal and any other BMJ products and to exploit all rights, as set out in our licence.

The Submitting Author accepts and understands that any supply made under these terms is made by BMJ to the Submitting Author unless you are acting as an employee on behalf of your employer or a postgraduate student of an affiliated institution which is paying any applicable article publishing charge ("APC") for Open Access articles. Where the Submitting Author wishes to make the Work available on an Open Access basis (and intends to pay the relevant APC), the terms of reuse of such Open Access shall be governed by a Creative Commons licence – details of these licences and which Creative Commons licence will apply to this Work are set out in our licence referred to above.

Other than as permitted in any relevant BMJ Author's Self Archiving Policies, I confirm this Work has not been accepted for publication elsewhere, is not being considered for publication elsewhere and does not duplicate material already published. I confirm all authors consent to publication of this Work and authorise the granting of this licence.

For peer review only

Drug Use in Street Sex worKers (DUSSK) study – results of a mixed methods feasibility study of a complex intervention to reduce illicit drug use in drug dependent female sex workers

Authors:

Dr Rita Patel*,[1,2] Dr Niamh M Redmond*,[1,2] Dr Joanna M Kesten,[1,2,3] Dr Myles-Jay Linton, [1,2,4] Dr Jeremy Horwood,[1,2,6] David Wilcox,[5] Jess Munafo,[5] Prof Joanna Coast,[1,2,4] Prof John Macleod,[1,2,6] Dr Nicola Jeal.[1,2,7]

*Joint first authors.

Author affiliations:

1 The National Institute for Health Research Applied Research Collaboration West (NIHR ARC West) at University Hospitals Bristol NHS Foundation Trust, Whitefriars, Lewins Mead, Bristol, UK, BS1 2NT

2 Population Health Sciences, Bristol Medical School, University of Bristol, Canynge Hall, 39 Whatley Road, Bristol, UK, BS8 2PS

3 The National Institute for Health Research Health Protection Research Unit in Evaluation of Interventions, University of Bristol, UK

4 Health Economics Bristol, Population Health Sciences, Bristol Medical School, University of Bristol, 1-5 Whiteladies Road, Bristol, UK, BS8 1NU

5 Avon & Wiltshire Partnership NHS Trust, Acer Unit, Blackberry Hill Hospital, Manor Road, Bristol, UK, BS16 2EW

6 Centre for Academic Primary Care, Population Health Sciences, Bristol Medical School, University of Bristol, Canynge Hall, 39 Whatley Road, Bristol BS8 2PS

7 Northern Devon Healthcare NHS Trust, Devon Sexual Health - North Devon, Barnstaple Health Centre, Vicarage Road, Barnstaple EX32 7BH

Corresponding author:

Dr Nicola Jeal (Nikki.Jeal1@nhs.net)

Word count:

Target Journal BMJ Open: Abstract: (297/300), Main: (4122/4000)

For peer review only

Abstract

Objectives

The majority of female street-based sex workers (SSWs) are dependent on illicit drugs and sell sex to fund their drug use. They typically face multiple traumatic experiences, starting at a young age, which continue through sex work involvement. Their trauma-related symptoms tend to increase when drug use is reduced, hindering sustained reduction. Providing specialist trauma care alongside drug treatment may improve treatment outcomes. Aims to (i) evaluate recruitment and retention of participants; (ii) examine intervention experiences and acceptability; and (iii) explore intervention costs using mixed methods feasibility study.

Setting

Female SSW charity premises in a large UK inner city.

Participants

Females aged 18 years or older, who have sold sex on the street and used heroin and/or crack cocaine at least once a week in the last calendar month.

Intervention

Female SSW-only drug treatment groups in a female SSW-only setting delivered by female staff. Targeted post-traumatic stress disorder (PTSD) screening then treatment of positive diagnoses with eye movement desensitization and reprocessing (EMDR) therapy by female staff from a specialist NHS trauma service.

Results

(i) Of 125 contacts 11 met inclusion criteria and provided informed consent, 4 reached the intervention final stage (ii) Service providers said working in collaboration with other services was valuable, the intervention was worthwhile and had a positive influence on participants. Participants viewed recruitment as acceptable and experienced the intervention positively. The unsettled nature of participant's lives was a key attendance barrier. (iii) The total cost of the intervention was £11,710, with staff costs dominating.

Conclusions

Recruitment and retention rates reflected study inclusion criteria targeting women with the most complex needs. Two participants received EMDR demonstrating that the three agencies working together was feasible. Staff heavy costs highlight the importance of supporting participant attendance to minimise per participant costs in a future trial.

For peer review only

Ethics approval

South West - Frenchay Research Ethics Committee (REC reference: 17/SW/0033; IRAS project ID: 220631). UK HRA approval was on 03/04/2017.The University of Bristol Research Enterprise and Development department sponsored this study (RED reference: RG2756).

Strengths and limitations of this study

- The novel intervention integrates a trauma-focussed treatment approach in order to reduce drug use in a challenging drug treatment population
- The intervention was delivered by specialists, reflecting the skills and experience required to appropriately manage the complex needs of the study population
- PPI formed an important part of the study methodology and informed each stage
- Recruitment took place over several months within an agency trusted and used daily by the study population to allow familiarisation with the researchers and multiple opportunities to participate
- This feasibility study design and methodology were not able to examine intervention effectiveness or cost effectiveness

Keywords:

sex workers, feasibility study, qualitative research, cost analysis, trauma treatment, drug dependency

For peer review only

Introduction

Most female street-based sex workers (SSWs) in the UK use heroin and/or crack cocaine.[1-3] Their drug dependency keeps them entrenched in a “work-score-use cycle”,[4 5] which contributes to the morbidity and social instability typically seen in this group.[6]

Despite their drug treatment needs, drug dependent SSWs have poorer outcomes from drug treatment services compared to other service users,[7 8] sometimes due to stigma associated with their street sex work.[9] Previous SSW-focussed interventions aiming to reduce drug use have used educational,[10 11] substitute prescribing-based[12 13] and psychological[14] approaches but none robustly demonstrated a positive effect in reducing drug use.[15]

Poor mental health is a significant problem among SSWs.[16-18] Many have experienced multiple adversities in early life and during their involvement in sex work,[5 16 19] which exposes women to further risk of significant trauma.[16 17] Consequently, many SSWs are affected by post-traumatic stress disorder (PTSD).[16 17] Trauma symptoms, which often recur when drug use is reduced, may motivate a return to drug use.[20] Individual trauma-focused therapy alongside drug treatment may provide the best outcomes for reductions in drug use.[21-23] However to date, there is no robust evidence to demonstrate the impact of an integrated trauma-focussed treatment approach in reducing drug use amongst female drug dependent SSWs.

In collaboration with SSWs and service providers we developed a novel intervention, informed by existing research[9 15] to address the unique and complex combination of drug and trauma treatment needs of female drug-dependent SSWs. The intervention proposes an integrated care pathway through an innovative multi-agency partnership.[24] We report here the results of the DUSSK (Drug Use in Street Sex worKers) feasibility study, which aimed to (i) evaluate the recruitment and retention of SSWs to the intervention; (ii) examine the experience and acceptability of the intervention for participants and service providers; and (iii) explore costs to service providers associated with the intervention.

For peer review only - http://bmjopen.bmj.com/site/about/guidelines.xhtml

Methods

Study design, setting and eligibility

Detailed methods are described in the published protocol.[24] This mixed methods feasibility study took place in a UK inner city setting. Females aged 18 years or older, who sold sex on the street at least weekly in the last calendar month and used heroin and/or crack cocaine at least once a week in the last calendar month, were eligible to participate.[24] The intervention was delivered at SSW charity premises, which supplied support, health and advocacy services.[24]

Recruitment

The recruitment target for this feasibility study was 30 women.[24] Local organisations that SSWs were known to access were provided with study promotional materials. One of three researchers (JMK, NJ, SR) attended an SSW support and advocacy charity, at least twice a week, to directly approach potential participants; alternatively, interested SSWs could telephone researchers. This approach meant that women were potentially approached and counted as contacts several times during recruitment. Eligibility screening was conducted face-to-face or via telephone. Women gave fully informed, written consent to participate in the study and baseline data were also collected at the time of recruitment. To maintain safety and confidentiality, each participant provided details of acceptable ways in which to be contacted. Screening data were retained and remained anonymised for those not recruited.

Patient and Public Involvement (PPI)

Women with experience of street sex work and drug-dependency contributed to the study design, processes, documentation and intervention development. Recommendations from PPI meetings were implemented and resulted in direct changes to the study processes to support recruitment, participation and encourage adherence (described below).

The intervention

The intervention consisted of SSW only drug treatment groups, targeted screening for PTSD symptoms (one-to-one clinical interview and PTSD checklist- PCL5)[25] and, if positively diagnosed, one-to-one EMDR therapy, all delivered by female staff through a collaboration between three service providers (National Health Service (NHS) trauma services, the SSW and drug treatment charities). The intervention was designed so participants were initially invited to attend a weekly 'Getting started'

For peer review only - http://bmjopen.bmj.com/site/about/guidelines.xhtml
For peer review only

drug treatment group to reduce fear and anxiety about engaging in a group setting and get used to the format and level of disclosure expected. Participants were to progress to a 'Preparation for recovery' drug treatment group which focused on people's barriers to motivation for change, examining pros and cons of drug use and exploring triggers for using drugs to enable participants to achieve a level of drug use stability. When group facilitators judged participants were achieving drug use stabilisation, and they had attended three sessions consecutively, they were offered screening for PTSD symptoms by a female clinical psychologist. Those experiencing PTSD symptoms were invited to attend five PTSD 'Stabilisation' group sessions, facilitated by the same female clinical psychologist, to equip participants with the skills to self-soothe and reorientate in preparation for the one-to-one EMDR treatment. Once all 'Stabilisation' group sessions had been completed, the clinical psychologist assessed participants for readiness for one-to-one EMDR sessions and if eligible, participants progressed to a course of 12 sessions with the clinical psychologist on a weekly, or fortnightly, basis. Trauma treatment (PTSD screening, stabilisation groups and one-to-one) ran in parallel to the drug treatment groups, Figure 1 (red boxes) shows the planned flow of participants through the study.

The intervention proceeded as described in the protocol paper [24] with the following changes (bulleted below):

- Participants were encouraged to attend all sessions with the offer of car lifts, bus tickets and taxis, in addition to the planned weekly phone calls and texts, by the staff at partner agencies.

'Getting started' and 'Preparation for recovery' drug treatment groups

- Retendering resulted in a change of drug group service provider which, along with low numbers recruited, resulted in the drug groups merging into a single open drug treatment 'Getting started' group.
- Attendance at three consecutive sessions was required to move onto the next group, instead of attendance at any four.
- Sandwiches were supplied prior to the single drug group to support attendance from the 14th session onwards.
- The number of sessions continued beyond those initially planned due to delays in PTSD screening (see below).
- Poor attendance, the participant's unstable behaviour and the intervention running for longer than planned (due to the retendering of drug services) affected the drug group facilitators' ability to deliver structured content. An art worker was included in 4 sessions to maintain participant's interest and engagement with sessions.

For peer review only - http://bmjopen.bmj.com/site/about/guidelines.xhtml

- PTSD screening was halted from the 29th session onwards, due to the limited remaining study time to complete the intervention, meaning the drug group became closed and only included PTSD screened participants.

Screening for PTSD

- Delays occurred to both the organisation of the PTSD screening and PTSD stabilisation group set up due to lack of capacity and delays in NHS trauma service staff recruitment. Therefore, some participants had long gaps between recruitment and PTSD screening and subsequent stabilisation group sessions.

PTSD 'Stabilisation' group

- Incentives of £10 vouchers per session were offered for attendance (mandatory sessions).

One-to-one EMDR therapy for PTSD

- Some sessions were scheduled in a private rented room (due to availability issues) in a local community centre and not at the SSW charity premises.
- Two participants were offered weekly one-to-one extended stabilisation sessions (8 maximum) with the clinical psychologist rather than EMDR therapy as this was deemed the most appropriate treatment.

Data collection methods

Sample size and quantitative data collection

A formal sample size calculation was not conducted as the aim was to assess feasibility.[24] Self-reported levels of illicit drug use, involvement in SSW, completion of PTSD Checklist PCL5[25] and demographics were collected at the time of consent. Attendance registers were taken at the start of each group or one-to-one session by the facilitator(s).

Qualitative data collection

With participants' verbal consent one non-participant observation of a drug group was conducted to understand delivery, examine interactions and intervention experiences, with brief notes taken during the group.[26]

In-depth semi-structured interviews were conducted with participants and service providers either face-to-face or by telephone. Participants were interviewed after intervention completion or study

For peer review only - http://bmjopen.bmj.com/site/about/guidelines.xhtml
For peer review only

drop out. Consent to contact participants regarding interviews was sought at recruitment. Additional written or audio recorded verbal, informed consent was obtained prior to all interviews. Interviews explored views and experiences of the intervention and how to improve acceptability. Participants received a £20 high street shopping voucher for taking part. Most service provider interviews were conducted at the end of the intervention period and also sought to understand operational issues, inter-agency working and intervention delivery.

Economic data collection

Resource use information (2018 £GBP) was collected prospectively by the agencies. Data collection focussed on four categories: Staff time, Facilities, Travel (provider funded staff and patient transport), and Materials.

Data analysis

The integration of qualitative and quantitative data used the established 'following a thread' technique [27] where key themes were traced using all data sets.

Statistical analysis

Descriptive statistics (using Stata version14) were used to monitor recruitment and retention via CONSORT statement[28] style flowcharts, and to examine participant demographics, questionnaire responses and attendance.

Qualitative analysis

Interviews were conducted by JMK and NJ, and the audio files transcribed, anonymised and checked for accuracy. QSR NVivo 10 software was used to perform inductive thematic analysis[29] using constant comparison techniques.[30 31] A preliminary coding framework was developed by JMK and discussed with the multidisciplinary research team JH and NJ to ensure credibility and confirmability.

Cost analysis

Costs to the service provider were examined and summarised separately for each of the four intervention components. Staff costs were calculated using salaries and on-costs or generated using standard unit cost data available for health and social care professionals.[32] Facility costs were calculated based on similar space rental options. Total cost, total cost per eligible participant, total cost per session held, and total cost per session per eligible participant were calculated for each

For peer review only - http://bmjopen.bmj.com/site/about/guidelines.xhtml

intervention component. Cost data were tabulated using principles of heat-map methodology, where colour lightness is used to communicate the magnitude of different costs.[33 34]

For peer review only

For peer review only - http://bmjopen.bmj.com/site/about/guidelines.xhtml

Results

Recruitment was from November 2017 to March 2018 with the intervention delivered until December 2018.

(i) Recruitment and retention

Recruitment Process

Approximately 400 flyers and nine posters were distributed. Potential participants were spoken to by three researchers at 37 three-hour 'drop-in' sessions at the SSW charity. Of 125 contacts made with women, 84 declined screening. Reasons for decline included being too busy (n=13), not interested (n=11) or reporting not currently street sex working (n=10). Fourteen approaches were reported as repeat approaches, with seven reporting previous recruitment and four previous screening (as the researchers recruited on different weekdays and screening data was anonymous). Figure 1 details the flow of screened and recruited participants through the intervention.

Of 41 women screened, 11 were eligible and consented to participate, three were eligible but unable to give consent (two were too distressed and one had health issues preventing participation). Of the 27 ineligible women, two did not fully complete the screening questions. The main reasons for exclusion related to ineligible frequency of drug use and/or sex work. Table 1 shows the range of days since responders last street sex worked or had taken heroin/crack by those recruited and not recruited.

Of 11 participants consenting to be invited for qualitative interviews, five were uncontactable. Seven interviews were conducted with six participants (six face-to-face and one via telephone); four interviewed participants received all components of the intervention. Ten service provider interviews were conducted with representatives from the drug treatment service (n=4), the trauma service (n=2), and the SSW charity (n=4). Table 2 details quotes from the interviews to support the main results described below.

Acceptability of recruitment

The recruitment process was described as acceptable by most participants and service providers. Face-to-face recruitment was experienced as confidential, with participants reporting receiving clear explanations of the study. Recruitment over five months within the SSW charity offered multiple opportunities to participate and was acceptable. Most service providers interviewed reflected that if

For peer review only - http://bmjopen.bmj.com/site/about/guidelines.xhtml

study inclusion criteria were broadened to include less regular drug use, participants with more lifestyle stability could have been recruited, and whom may have found the intervention easier to engage with. However, some felt that creating groups with diverse levels of drug use could have negative consequences for example, risk of relapse for those who had reduced their drug use.

Group attendance

All 11 consented participants were invited to attend drug treatment groups. However, participants attendance varied throughout the study, with participants sometimes arriving late or leaving early (Table 3). Four attended 30-76% of sessions compared to 7 attenders who attended only 0-18% of sessions. The five most frequent attenders were invited to PTSD screening of which four attended and were all found to have symptoms of PTSD. All four PTSD screened participants attended 20-100% of the stabilisation groups with the clinical psychologist. At the end of the stabilisation groups, two participants were deemed suitable for EMDR therapy by the clinical psychologist and two were offered extended stabilisation sessions. All four participants attended some one-to-one sessions (Table 3) but missed at least two consecutive trauma treatment appointments with the clinical psychologist and had to withdraw from the sessions. However, all participants were referred to further mental health services and one participant was also referred to mainstream drug services.

Facilitators to attendance

Service providers across all partner agencies sent reminders to participants, which were described as helpful and appreciated by participants. One SSW charity service provider played a vital role in encouraging attendance through reminding participants to attend, arranging transport (taxi, bus or driving participants to sessions) and helping participants prepare for the intervention. Provision of sandwich lunches before the groups was seen by service providers and participants as helpful for encouraging attendance, facilitating a relaxed start to groups and supporting concentration. Vouchers were also viewed as encouraging attendance by participants and service providers.

Barriers to attendance

The unsettled nature of participant's lives was perceived as an attendance barrier and was underpinned by problematic drug use, poor adherence to opioid substitution therapy, sex work, tiredness and poor mental health. Arguments between participants, a lack of readiness to confront issues with drugs and trauma and an absence of social support were described as making attendance difficult. Delays to screening referral and trauma treatment were also reported to negatively affect participants' motivation.

For peer review only - http://bmjopen.bmj.com/site/about/guidelines.xhtml

(ii) Experience and acceptability of the intervention

Initial impressions

All participants perceived the intervention as valuable and welcome. Common reasons given by participants for taking part included the opportunity for 'change', greater stability and valuing the opportunity to combine mental health and drug treatment. Service providers viewed the intervention as a novel opportunity for 1) SSWs to receive mental health treatment while continuing to use drugs and 2) to address the barriers to mainstream drug treatment. Some service providers highlighted the challenge for participants to process trauma while continuing to use drugs and potential risks of sex working when receiving trauma treatment.

Service providers' views on the intervention

The drug group facilitators described enjoying delivering the groups and building good relationships with participants. However, they described some drug sessions as intense and difficult to manage due to participants' distress, accounts of trauma and chaotic behaviour. The need for appropriate support and supervision of facilitators was highlighted as a requirement to manage these challenges.

A clinical psychologist suggested that without the 're-traumatising' effects of street sex work, the effectiveness of the trauma processing in the trauma treatment might be enhanced. Service providers also proposed extending the stabilisation work to develop the effectiveness of the trauma treatment and recommended the intervention offer alternatives to EMDR to suit individual participants' needs.

Service providers said working in partnership with other specialist services to deliver the intervention was valuable, there was mutual respect and good communication between staff. It was suggested it would have been useful to have collaborative, regular case-review meetings between the services to assess the progress and needs of the participants and enhance the communication channels.

Participants views on the intervention

Participants described generally positive intervention experiences. They described forming meaningful relationships with the drug group facilitators and clinical psychologist. They liked that the groups were female and sex worker-only, they knew other participants already and could speak openly about, and relate to, one another's experience of trauma, drug use and street sex work. Participants also valued that the intervention was delivered at SSW charity premises, which was liked for its familiarity, safety, comfort, convenience and freedom from judgement and shame. The day of the

For peer review only - http://bmjopen.bmj.com/site/about/guidelines.xhtml

week, time and frequency of sessions, drug group session length and group size were also acceptable to most participants and service providers. These factors overcame some of the barriers participants highlighted to attending mainstream drug services.

Strengths of the intervention

Through the intervention, participants reflected on their need to address their trauma and drug use. Some acknowledged that they were not ready to address their trauma but aspired to this in future, having had positive experiences of therapy during the intervention. Participants attributed improved wellbeing, coping strategies and perceptions of self-worth to the intervention. One participant was seen less on the SSW outreach van (an indicator of sex working) and, significantly, stopped using her working name, signifying 'taking back ownership of who she is'. One drug group facilitator felt the flexibility of the study intervention was able accommodate participants' unstable lives and level of trauma that would have prevented them from complying with the rules of conduct in mainstream drug services and thus prevented them from receiving treatment to address their needs. They also felt that an additional positive feature of the intervention was participants being able to discuss their sex work, due to the female SSW only membership of the groups.

Participation in the intervention was described by SSW charity service providers and two participants as supporting and empowering the participants to engage with clinical and support services to address their needs. Another participant felt she used the SSW charity services less now because she needed less support.

(iii) Cost analysis

The total cost of the intervention was £11,710, with staff costs being the largest component (Table 4). The most expensive component of the intervention was the 'Getting started' sessions (which totalled £6,842). Despite having the second to lowest sub-total cost across the intervention (£1,014), the stabilisation groups had the highest cost per session held (£203). Although the one-to-one sessions had the lowest cost per session held (£103), the larger number of sessions at this point resulted in this section having the highest cost per eligible participant (n=4, £724). Trauma screening had the lowest cost per eligible participant (n=5, £191)

Fidelity

The intervention was broadly delivered as intended incorporating suggested planned changes to the protocol, it was more flexible and less linear than originally planned.[24] Delays in PTSD screening meant that there was only a single drug group, which continued for longer than originally planned.

For peer review only - http://bmjopen.bmj.com/site/about/guidelines.xhtml

Discussion

Summary of findings

This study used a mixed methods approach to investigate the feasibility and acceptability of a novel, complex intervention to reduce illicit drug use in female drug-dependent SSWs. We demonstrated that drug-dependent SSWs could maintain attendance at female SSW-only drug group sessions and the integrated trauma-focussed treatment approach, in a trusted and supportive environment, with intensive support from SSW charity staff. Recruitment was lower than anticipated, with four participants PTSD screened and whom met criteria for PTSD. They progressed through to the final stage of the intervention, all four participants were ultimately able to access mental health services and one began the process of accessing mainstream drug services. Participants and service providers mostly experienced the recruitment process, the intervention and delivery mechanisms (especially the SSW-only environment) positively. Managing SSW trauma proved challenging for participants, drug group facilitators and the clinical psychologist and resulted in the need for intervention refinement. Attendance and adherence barriers primarily related to the issues the intervention sought to address, namely problematic drug use, sex work, and poor mental health, rather than the acceptability of the intervention itself. The total cost of the intervention was £11,710, with staff costs dominating.

Strengths and limitations of this study

Strengths of our study were that recruiting researchers also conducted the interviews, which may have facilitated a rapport with participants and supported more open and honest reflections. Secondly, all aspects of the intervention were delivered by specialists which was necessary for this high-risk group with multiple complex co-morbidities. Thirdly, PPI formed an important part of the study and informed each stage. Fourth, recruitment over several months with multiple approaches allowed SSWs many opportunities to take part. This approach took account of the rapidly changing lives of SSWs resulting in changing eligibility status as well as allowing time for them to become familiar with the researchers. Finally, recruitment within a trusted agency may have had a positive influence on recruitment.

This feasibility study provides only preliminary information on the intervention performance and cost and does not examine the effectiveness or potential for reducing costs in other parts of the health service or wider society. Delays due to changes to service provision are likely to have adversely influenced study recruitment and retention, with delays resulting in higher service costs, but reflect the real-life issues facing multi-agency work.

For peer review only - http://bmjopen.bmj.com/site/about/guidelines.xhtml

Comparison with other research

This feasibility study is the first, to our knowledge, to address previously highlighted barriers to effective drug treatment for SSWs.[15 22 23] Through incorporating female SSW-only drug groups alongside an intervention with specialised trauma treatment, delivered in a female SSW-only setting by female staff[22 24] we showed how an integrated treatment approach in this complex vulnerable group could feasibly be implemented and delivered, with small changes to the intervention.

This study is the first interventional study to employ clinical staff from a specialist trauma service to deliver EMDR to address trauma symptoms as part of the drug treatment process for SSWs. These women have been found to have high levels of poor mental health,[16 18] particularly trauma,[5 16 17] which contributes to poor drug treatment outcomes.[7 8] The intervention took account of SSWs frequent experience of abuse and violence,[35 36] and recommendations for female-only trauma-focussed drug treatment interventions[22 37 38] for treatment of PTSD and long term drug use reduction. Some of the characteristics the intervention sought to address presented as barriers to attendance and retention, however these are common in studies trying to effect behaviour changes within vulnerable groups.[23 39]

Previous SSW-focussed interventions aiming to reduce drug use[10-13 14] were unable to demonstrate strong evidence of a positive effect[15] suggesting the need for a novel approach with evidence of efficacy assessed through a robust methodological approach. The highlighted barriers to attendance, engagement and delivery of the intervention are in keeping with other studies[15 23 39] but indicate that further changes to the DUSSK feasibility study design are likely to be required in future studies.

Conclusion and implications for service provision and research

This study sought to explore the feasibility of delivering a novel complex intervention to a challenging population with high levels of unmet need. Inclusion criteria were informed by PPI, clinical expertise and academic literature. They targeted women who were likely to benefit the most from a trauma-based intervention but whose drug dependency and chaotic lives made them challenging study participants to recruit and retain. However, all four of the participants screened for PTSD were diagnosed, revealing the unmet need for trauma treatment. Though unsurprising, the severity of trauma disclosed by SSWs proved unexpectedly challenging for service providers.[22 37 38] Further data to understand the extent and severity of PTSD in SSWs is recommended to inform service provision.

For peer review only - http://bmjopen.bmj.com/site/about/guidelines.xhtml

Overall, the experiences described by those receiving the intervention, suggest it is an acceptable approach to reducing SSWs drug use.

The three services found the intervention valuable and were able to work together effectively despite setbacks such as changing contracts and service pressures, and suggested closer working could improve intervention delivery.

Intervention costs were driven up by poor participant attendance, though staff pressures and the retendering process increased the length (and cost) of the intervention period. However, decreased SSW use of health services, the criminal justice system and impacts of criminal activity on wider society may justify its adoption if future trials demonstrate intervention effectiveness in reducing drug use.

In order to support future interventional trials in this important field where there are few effectiveness studies we recommend the following study refinements for consideration.

1. The intervention could include women with more stability in their lives to increase recruitment and retention.
2. Regular meetings throughout the study enabling all service providers involved in intervention delivery to express concerns and seek to understand participants needs from the perspective of different professionals so there is effective multi-agency support for individual participants where needed.
3. Training for all involved staff in managing the disclosure of trauma.
4. Support and encouragement for participant engagement through provision of transport to and refreshments prior to treatment sessions
5. Intervention flexibility and responsiveness in offering trauma-focussed alternatives to EMDR which may be more suitable for individual participants needs.
6. An extended trauma therapy programme to accommodate the complexity of SSW needs.

For peer review only

Additional information

Acknowledgements

The authors are extremely grateful to all the women who have participated in the study; all service providers and all other staff whose participation made this study possible. They would like to thank the PPI group for their time, thoughts and suggestions. We are grateful to the following organisations and individuals who have helped with the study for their time, expertise and support: Developing Health and Independence, Bristol Drugs Project, Avon & Wiltshire Mental Health Partnership NHS Trust and One25 charity, Katie Warner, Lucy Pettler, Rosie Davies, Maggie Telfer, Elinor Griffiths, Gill Nowland, Sophie Ramsden, Elaine Driver, Anna Smith, Rhea Warner, Jennifer Riley, Tracey Tudor, Madeline Saunders, Charlotte Hignell, Sophie Banks, Jane Bowman, Sarah Shatwell, Katrina Turner, Hasina Downie, Jo Daniels and Louisa Chowen.

Author Contributions

NJ, NMR, JH, JMK, RP, DW, JM, JMa, MJL and JC are responsible for the study design, collection of data and analysis. NMR, RP, NJ and JH are responsible for study management and coordination. JMK and NJ conducted the interviews. JMK led the qualitative analysis in collaboration with NJ and JH. RP conducted the quantitative analysis. MJL, RP and JC conducted the costing study. NMR, RP, JMK, MJL, JC, NJ and JH drafted the paper. All authors read, commented on and approved the final manuscript.

Funding

The research is supported by a National Institute for Health Research (NIHR) Clinic Trials Fellowship awarded to NJ (NIHR-CTF-2016-05-07), the National Institute for Health Research Collaboration for Leadership in Applied Health Research and Care West (NIHR CLAHRC West P324), now recommissioned as NIHR Applied Research Collaboration West (NIHR ARC West) and Research Capability Funding awarded by University Hospitals Bristol NHS Foundation Trust (RCF 2016-17-18). JMK is partly funded by NIHR Health Protection Research Unit in Evaluation of Interventions. The views expressed are those of the authors and not necessarily those of the NIHR or the Department of Health and Social Care.

Competing interests

None declared

Data availability statement

All data relevant to the study are included in the article.

For peer review only - http://bmjopen.bmj.com/site/about/guidelines.xhtml

For peer review only

For peer review only - http://bmjopen.bmj.com/site/about/guidelines.xhtml

References

1. Cusick L, Martin A, May T. Vulnerability and involvement in drug use and sex work. Home Office research study 268. London: Home Office, 2003.
2. Deering KN, Kerr T, Tyndall MW, et al. A peer-led mobile outreach program and increased utilization of detoxification and residential drug treatment among female sex workers who use drugs in a Canadian setting *Drug & Alcohol Dependence* 2011;113(1):46-54.
3. Morris D, Lemus H, Wagner K D, et al. Factors associated with pathways toward concurrent sex work and injection drug use among female sex workers who inject drugs in northern Mexico. *Addiction* 2013;108:161-70.
4. Jeal N, Salisbury C, Turner K. The multiplicity and interdependency of factors influencing the health of street-based sex workers: a qualitative study. *Sex Transm Infect* 2008;84(5):381-5. doi: 10.1136/sti.2008.030841 [published Online First: 2008/07/04]
5. Gorry J, Roen K, Reilly J. Selling your self? The psychological impact of street sex work and factors affecting support seeking. *Health Soc Care Community* 2010;18(5):492-9. doi: 10.1111/j.1365-2524.2010.00925.x [published Online First: 2010/06/30]
6. Jeal N, Salisbury C. Health needs and service use of parlour-based prostitutes compared with street-based prostitutes: a cross sectional survey. *BJOG* 2007;114:875-81.
7. Burnette M LE, Ilgen M, Frayne S, Mayo J, Weitlauf J. Prevalence and health correlates of prostitution among patients entering treatment for substance use disorders. *Archives of General Psychiatry* 2008;65(3):337 - 44.
8. Burnette M SR, Timko C, Ilgen M. Impact of substance-use disorder treatment on women involved in prostitution: substance use, mental health and prostitution one year after treatment. *Journal of Studies on Alcohol and Drugs* 2009;70:32-40.
9. Jeal N, Macleod J, Salisbury C, et al. Identifying possible reasons why female street sex workers have poor drug treatment outcomes: a qualitative study. *BMJ Open* 2017;7(3):e013018. doi: 10.1136/bmjopen-2016-013018 [published Online First: 2017/03/25]
10. Sherman SG, German D, Cheng Y, et al. The evaluation of the JEWEL project: An innovative economic enhancement and HIV prevention intervention study targeting drug using women involved in prostitution. *Aids Care-Psychological and Socio-Medical Aspects of Aids/Hiv* 2006;18(1):1-11. doi: 10.1080/09540120500101625
11. Surratt HL, Inciardi JA. An effective HIV risk-reduction protocol for drug-using female sex workers. *J Prev Interv Community* 2010;38(2):118-31. doi: 10.1080/10852351003640732 [published Online First: 2010/04/15]
12. Gunne LM, Gronbladh L, Petersson S. [Methadone treatment in the prevention of AIDS. Heroin-dependent prostitutes are an important target]. *Lakartidningen* 1986;83(49):4194-6. [published Online First: 1986/12/03]
13. Litchfield J, Maronge A, Rigg T, et al. Can a targeted GP-led clinic improve outcomes for street sex workers who use heroin? *The British journal of general practice : the journal of the Royal College of General Practitioners* 2010;60(576):514-6. doi: 10.3399/bjgp10X514774 [published Online First: 2010/07/03]
14. Yahne CE, Miller WR, Irvin-Vitela L, et al. Magdalena Pilot Project: motivational outreach to substance abusing women street sex workers. *J Subst Abuse Treat* 2002;23(1):49-53. [published Online First: 2002/07/20]
15. Jeal N, Macleod J, Turner K, et al. Systematic review of interventions to reduce illicit drug use in female drug-dependent street sex workers. *BMJ Open* 2015;5(11):e009238. doi: 10.1136/bmjopen-2015-009238 [published Online First: 2015/11/20]
16. El-Bassel N, Schilling R, Irwin K, et al. Sex trading and psychological distress among women recruited from the streets of Harlem. *American Journal Public Health* 1997;87:66-70.
17. Chukakov B, Ilan K, Belmaker RH, et al. The motivation and mental health of sex workers. *Journal of Sex & Marital Therapy* 2002;.28(4):pp. doi: 10.1080/00926230290001439

For peer review only - http://bmjopen.bmj.com/site/about/guidelines.xhtml

18. Roxburgh A, Degenhardt L, J C, et al. Drug Dependence and Associated Risks Among Female Street-Based Sex Workers in the Greater Sydney Area, Australia. *Substance Use & Misuse* 2008;43(8-9):1202-17.
19. Mellor R, Lovell A. The lived experience of UK street-based sex workers and the health consequences: an exploratory study. *Health promotion international* 2012;27(3):311-22. doi: 10.1093/heapro/dar040 [published Online First: 2011/07/06]
20. Reynolds M, Mezey G, Chapman M, et al. Co-morbid post-traumatic stress disorder in a substance misusing clinical population. *Drug and alcohol dependence* 2005;77(3):251-58. doi: https://doi.org/10.1016/j.drugalcdep.2004.08.017
21. Cloitre M, Courtois CA, Charuvastra A, et al. Treatment of complex PTSD: Results of the ISTSS expert clinician survey on best practices. *Journal of Traumatic Stress* 2011;24(6):615-27. doi: 10.1002/jts.20697
22. Roberts NP, Roberts PA, Jones N, et al. Psychological therapies for post-traumatic stress disorder and comorbid substance use disorder. *Cochrane Database Syst Rev* 2016;4(4):CD010204. doi: 10.1002/14651858.CD010204.pub2 [published Online First: 2016/04/05]
23. Bailey K, Trevillion K, Gilchrist G. What works for whom and why: A narrative systematic review of interventions for reducing post-traumatic stress disorder and problematic substance use among women with experiences of interpersonal violence. *Journal of Substance Abuse Treatment* 2019;99:88-103. doi: 10.1016/j.jsat.2018.12.007
24. Jeal N, Patel R, Redmond NM, et al. Drug use in street sex workers (DUSSK) study protocol: a feasibility and acceptability study of a complex intervention to reduce illicit drug use in drug-dependent female street sex workers. *BMJ Open* 2018;8(11):e022728. doi: 10.1136/bmjopen-2018-022728 [published Online First: 2018/11/06]
25. Bovin MJ, Marx BP, Weathers FW, et al. Psychometric properties of the PTSD Checklist for Diagnostic and Statistical Manual of Mental Disorders-Fifth Edition (PCL-5) in veterans. *Psychol Assess* 2016;28(11):1379-91. doi: 10.1037/pas0000254 [published Online First: 2016/11/04]
26. Emerson RM, Fretz RI, Shaw LL, et al. Writing ethnographic fieldnotes. Chicago and London: University of Chicago Press 1995.
27. Moran-Ellis J, Alexander VD, Cronin A, et al. Triangulation and integration: processes, claims and implications. 2006;6(1):45-59. doi: 10.1177/1468794106058870
28. Schulz KF, Altman DG, Moher D, et al. CONSORT 2010 statement: updated guidelines for reporting parallel group randomised trials. *BMJ* 2010;340:c332. doi: 10.1136/bmj.c332 [published Online First: 2010/03/25]
29. Braun V, Clarke V. Using thematic analysis in psychology. *Qualitative Research in Psychology* 2006;3(2):77-101.
30. Glaser B, Strauss A. The Constant Comparative method of Qualitative Analysis. In the Discovery of Grounded Theory: Strategies for Qualitative Research Chicago: Aldine 1967:101-158.
31. Charmaz K. Constructing grounded theory: a practical guide through qualitative analysis. London: Sage Publications, Inc 2006.
32. Curtis LB, A. . Unit Costs of Health and Social Care University of Kent, Canterbury: Personal Social Services Research Unit 2018.
33. Kirk A. Data Visualization: a successful design process: Packt Publishing Ltd 2012.
34. Iliinsky N, Steele J. Designing data visualizations: Representing informational Relationships: O'Reilly Media, Inc. 2011.
35. Ulibarri MD, Hiller SP, Lozada R, et al. Prevalence and characteristics of abuse experiences and depression symptoms among injection drug-using female sex workers in Mexico. *J Environ Public Health* 2013;2013:631479. doi: 10.1155/2013/631479 [published Online First: 2013/06/06]

For peer review only - http://bmjopen.bmj.com/site/about/guidelines.xhtml

36. Monica D. Ulibarri, Steffanie A. Strathdee, Remedios Lozada, et al. Prevalence and Correlates of Client-Perpetrated Abuse Among Female Sex Workers in Two Mexico–U.S. Border Cities. *Violence Against Women* 2014;20(4):427– 45.
37. Greenfield SF, Brooks AJ, Gordon SM, et al. Substance abuse treatment entry, retention, and outcome in women: a review of the literature. *Drug and alcohol dependence* 2007;86(1):1-21. doi: 10.1016/j.drugalcdep.2006.05.012 [published Online First: 2006/06/09]
38. Wiechelt SA, Shdaimah CS. Trauma and Substance Abuse Among Women in Prostitution: Implications for a Specialized Diversion Program. *Journal of Forensic Social Work* 2011;1(2):159-84. doi: 10.1080/1936928x.2011.598843
39. Bailey K, Trevillion K, Gilchrist G. "We have to put the fire out first before we start rebuilding the house": practitioners' experiences of supporting women with histories of substance use, interpersonal abuse and symptoms of post-traumatic stress disorder. *Addiction Research & Theory* 2019:1-9. doi: https://doi.org/10.1080/16066359.2019.1644323

For peer review only

Figure 1 Flow of participants through the DUSSK study

For peer review only

For peer review only - http://bmjopen.bmj.com/site/about/guidelines.xhtml

Table 1 Characteristics of screened women

	Screened not recruited (N=30)		Screened and recruited (N=11)	
	N (%) *	Median (range)	N (%) *	Median (range)
Female	27 (90)		11 (100)	
Age	23 (77)	37 (26-55)	11 (100)	38 (23-53)
Ever sold sex on the street?				
Yes	26 (87)		11 (100)	
No	1 (3)		-	
How many days since last worked on the street?	23 (77)	60 (1-2920)	11 (100)	7 (1-28)
How often usually sell sex on street?				
Daily	6 (20)		3 (27)	
Weekly	5 (17)		8 (73)	
Less than weekly	16 (53)		-	
Ever used street drugs				
Yes	26 (87)		11 (100)	
No	1 (3)		-	
Ever used heroin	23 (77)		9 (82)	
Days since last used heroin	19 (63)	2 (0-731)	9 (82)	1 (0-6)
Ever used crack cocaine	23 (77)		11(100)	
Days since last used crack cocaine	21 (70)	2 (0-2922)		1 (0-4)
How often use heroin and/or crack cocaine?				
Daily	11 (37)		7 (64)	
Weekly	4 (13)		4 (36)	
Less than weekly	9 (30)		-	
Has an opioid substitute script	13 (43)		6 (55)	
Script type				
Buprenorphine/Subutex®	-		1 (9)	
Methadone	13 (43)		5 (45)	
Used other drugs:				
Alcohol	3 (10)		1 (9)	
Amphetamine	1 (3)		-	
Cannabis	5 (17)		5 (45)	
Spice	2 (7)		-	
MDMA (Ecstasy)	1 (3)			
Tramadol	1 (3)		-	
Sleeping tablets	-		1 (9)	
PCL5 score (possible range 0-80)	-		10 (91)	56 (43-73)

*N and % of those that provided data

For peer review only - http://bmjopen.bmj.com/site/about/guidelines.xhtml

Table 2 Qualitative quotes

Theme/Sub-theme	Quotes
**Recruitment and retention**	
**Acceptability**	*[Face-to-face recruitment] worked well, it wasn't intrusive, you weren't pushy, you know you blended in within the drop-in setting. So I think the women felt that if they did wanna buy into it they would, there was no pressure there. So I think that was done really sensitively.* Service provider 6<br><br>*It [recruitment] was very sort of like confidential and actually it was quite nice 'cause, yeah no one really knew what I was doing when I was doing summut, you know what I mean, which is – like it don't usually happen like that. Everyone knows what I'm doing all the time.* Participant 7
**Improvements**	*I think from a clinical point of view if you remove that criteria [sex work at least once a week in the last calendar month] and then of course there's more chance of getting people through to the finish line to be able to be ready for treatment at the end.* Service provider 4<br><br>*Actively drug using? Yes, that makes sense (…). If they've been able to bring that down themselves maybe another service would be better. Like, what this offered, it's specialistic in this. So if you was able to manage to a level yourself, maybe you don't need [the intervention]… I'm not sure, I think that would be an interesting conversation because if they could bring it down themselves, they'd probably be a lot more stable and a lot more reliable to actually get to the EMDR .* Service provider 7<br><br>*So I think if you were to extend the period of time and say 'Oh actually do you know if you've used within the last three months you can participate in the study and then someone who's three months abstinent or reducing from their street heroin use or their crack use is then exposed to somebody who's going no no no man I'm using up like a party every night'. There'd be that ethical thing within it but it would be nice to see the study opened up to a wider cohort.* Service provider 2
**Facilitators to attendance**	
Encouragement and support to attend	*I would say that I've been quite integral in regards to developing relationships with the women, contacting them for both their individual one to ones and stabilisation groups and also their Thursday DUSSK groups as well. So just keeping that contact going if they were coming in, in our drop-in service I would see them and then sort of give them reminders, did they want little welfare calls, that type of thing.* Service provider 6

For peer review only - http://bmjopen.bmj.com/site/about/guidelines.xhtml

Transport	*It was more of a focus thing where you know she [service provider 6] sort of like coached us as we went down, like you know keeping us like sort of aware of what we've got to be thinking of doing and making sure that, you know, there's nothing wrong.* Participant 7
Food provision	*I was turning up and I was like sort of god like hanging out for (…) that lunch. It was like, not the reason I was turning up but the main reason why I could (…). There is light at the end of the tunnel, you know you're gonna be nourished and fed.' You're gonna be able to concentrate as well.* Participant 7
**Barriers to attendance**	
Unstable lifestyles	*My mental problems, my drug use, everything, just my life, it gets in the way [of attendance].* Participant 1
Mental health	*They're so low resourced, they just don't have the distress tolerance to be able to cope with any more distress, they're already facing so much. Even things like their housing and threats of eviction.* Service provider 8<br><br>*My home life was getting a bit chaotic. My depression was getting really bad as well. So, yeah, and I was waiting for my antidepressants to work but they took a while. Yeah, it was just my depression, that's all.* My anxiety. Participant 6
Sex work	*If I've been working the night before there's no way I could have attended because I'm too tired, because you work all night.* Participant 4
Delays between treatment stages	*It took a little bit of a while and also for them to access their stabilisation groups then their one to ones. I think we may have lost some of the interest.* Service provider 6
**Experience and acceptability of the intervention**	
Initial impressions	*There aren't many services out there, which will offer individual, tailorised counselling and support to the women who have got dual diagnosis and you know mental health, drug misuse. So this study was unique and I think that's what we were all so passionate and so behind it because it was giving the women an opportunity.* Service provider 6
Reason for participating	*I just felt so alone and afraid and stuck and just needed to see if there was some way that I might be able to gain something so – really, if I'm willing to put myself out on the street and sell myself to a complete stranger, knowing that I might die, whatever, so it kind of … I felt I needed to understand why I needed to do this... So it's about me owning my power, and about not letting myself feel as shit about myself as I have done*. Participant 5

For peer review only

	The post-traumatic stress [treatment] is – is a way of like sort of detoxing your brain. So, you know finding a reason why you do these drugs (…) to like sort of be the reason for me to like say 'Well, I've got to stop now.' You know and get off it. Participant 7
Service providers views on the intervention	*I guess that people thought they weren't going to talk about their traumas [in the drug groups] but if somebody's been raped last night, they're going to need to talk about it, so we were here dealing with that stuff on the spot and then we didn't have no-one to go away and talk about it.* Service provider 10<br><br>*It's very hard to do trauma processing when women to some degree are being traumatised and then having to self-medicate against all of that and then you're trying to work on quite deep attachment developmental trauma stuff from a long time ago. (…) I'd say that trauma processing would be more successful with women who have maybe made a very strong commitment to stop [sex] working.* Service provider 8<br><br>*I would offer it [EMDR] as part of a – as a range of things that are offered…we'd say 'You can have EMDR, trauma focus CBT [Cognitive Behavioural Therapy] or narrative exposure therapy and you'd kind of match the person to what you thought they might be more suited to*. Service provider 8<br><br>*I think they had huge admiration for the workers at [SSW charity], and found them friendly and supportive, but…there wasn't a specific, I don't know, once a month structured 'let's talk about the women and how they've been in the month.* Service provider 9
Participants views of the intervention	*I enjoyed going down there. (...) We had a good laugh and learned something while we were doing it.* Participant 6<br><br>*With it being all woman and not mixed going to (mainstream drug treatment service provider) and doing groups where men are involved is like, I didn't really want to do it but here because it's all women and I know most of the women that come here, we've all been through it, hence why we all come here. So one way or another we've all been through something that we can all relate to.* Participant 3
Intervention characteristics	*It's [SSW charity] familiar and it's comfortable and it's safe.* Service provider 5

For peer review only - http://bmjopen.bmj.com/site/about/guidelines.xhtml

	The groups weren't too big, so you sort of – I knew the people that were coming to the groups which was better, so we'd sort of you know built up a rapport. Participant 4
Impacts of the intervention	*I'm just going to stop [drug use], I'm ready and I'm kind of already preparing for that, so it's kind of brought me to a close, and I mean that as well. Personally it's like, I'm ready, bring it on, I'm like do you know what I've been raped, I've been beaten I've stuck needles in myself(…) I'm done, I'm not playing this game anymore, I deserve better.* Participant 5<br><br>*It's just made (…) me realise I'm not just a, like, drug addict, sex worker. I'm a real person and I've got feelings and, you know, I've got potential. You know, yeah, they [service providers] build me up a lot.* Participant 6<br><br>*When she* [participant 6] *started with [name of intervention] study and she was coming to her Thursday [drug] groups, she didn't want to be associated with street sex working. So she said 'Can you call me (own name rather than working name)?' I could have cried (…). She was owning her own name and taking back ownership of who she is rather than somebody who was street sex working.* Service provider 6<br><br>*Their chaoticness. (…) To manage that in a [mainstream drug service] group setting would be difficult and I'm not sure how they would manage that. I just know how much regularly how they've turned up [to the intervention drug treatment groups] chaotic and they've turned up leaking out trauma. … I'm far from confident that they would be able to sit under them [mainstream drug service] rules enough to be a part of what it is for here[research study], due to the level of flexibility here and that they would be able to talk about what their problem is without mentioning what they do and that might make them vulnerable* Service provider 7
Fidelity	*I guess we were kind of thinking of it in a really linear sense, that the women would engage in the drug groups and then reduce their drug use to then move on to the next group and I'm not sure that that actually happened in reality.*<br>Service provider 5<br><br>*In the beginning we went in doing the same sort of work that we would do here [mainstream drug services], and it's getting them to look at their behaviour, and the consequences of it and stuff, and it didn't work with these women, it's too much, too direct.* Service provider 10

For peer review only - http://bmjopen.bmj.com/site/about/guidelines.xhtml

Table 3 Attendance and retention of participants

Participant	Open group Getting started: Eligible sessions	Attended sessions*	% Attended	Attended Trauma Screening	Closed group Getting started: Eligible sessions	Attended sessions	% Attended	5 mandatory Stabilisation group sessions: Attended sessions	% Attended	One-to-one sessions: Treatment	Eligible sessions**	Attended sessions*	% Attended	Referred to which services:
1	28	10	36	Yes	26	4	15	3	60	Ext. stabilisation	8[3]	1 (1)	13	Mental health
2	28	5	18	No										
3	28	1 (1)	4	NA										
4	27	8	30	Yes	26	11	42	5	100	EMDR	12[3]	4 (1) $	33	Mental health
5	25	3	12	NA										
6	25	19 (5)	76	Yes	26	15	58	4	80	EMDR	12[2]	8 (4) $	67	Mental health & mainstream drug
7	23	0	0	NA										
8	22	0	0	NA										
9	20	7 (2)	35	Yes	26	3	12	1	20	Ext. stabilisation	8[4]	0	0	Mental health
10	19	0	0	NA										
11	19	0	0	NA										
Total		53		4		33		13				13		

*N session participant arrived late/left early indicated in superscript round brackets; **N sessions cancelled due to non-attendance in square brackets; NA=Not actively invited to sessions; $ Includes one review session; Ext. stabilisation=Extended stabilisation, EMDR=Eye movement desensitization and reprocessing therapy

For peer review only - http://bmjopen.bmj.com/site/about/guidelines.xhtml

Table 4 Health Economics

	1. Getting started		2. Trauma screening		3. Stabilisation group		4. One-to-one sessions	
**Service description**								
Session lengths - range	90 - 120 minutes [1]		60 minutes		60 minutes		60 - 90 minutes [2]	
Number of sessions held - total	52		8		5		28	
Eligible participants[3] - total	n = 11		n = 5[4]		n = 4		n = 4	
Attendees per session - range	0 - 4		1		2 - 4		1	
**Costs**	**Sub-total £**	**£ per ppt**	**Sub-total £**	**£ per ppt**	**Sub-total £**	**£ per ppt**	**Sub-total £**	**£ per ppt**
**A. Staff**								
Service manager	£1,359.76	£123.61	£95.09	£19.02	£47.54	£11.89	£266.25	£66.56
Drug group facilitators[5]	£3,000.20	£272.75	£0.00	£0.00	£0.00	£0.00	£0.00	£0.00
Art worker	£123.98	£11.27	£0.00	£0.00	£0.00	£0.00	£0.00	£0.00
Clinical Psychologist	£0.00	£0.00	£636.00	£127.20	£600.00	£150.00	£2,040.00	£510.00
**B. Facilities**								
Space rental	£1,270.50	£115.50	£224.00	£44.80	£140.00	£35.00	£574.00	£143.50
**C. Travel**								
Transporting materials	£73.78	£8.44	£0.00	£0.00	£11.90	£2.98	£0.00	£0.00
Car lifts for service-users (petrol)	£38.72	£3.52	£2.24	£0.45	£3.36	£0.84	£1.20	£0.30
Public transport for participants	£70.20	£6.38	£0.00	£0.00	£3.90	£0.98	£3.90	£0.98
Taxis for participants	£55.00	£5.00	£0.00	£0.00	£62.20	£15.55	£11.00	£2.75
**D. Materials**								
Art supplies	£30.00	£2.73	£0.00	£0.00	£0.00	£0.00	£0.00	£0.00
Stationary	£0.00	£0.00	£0.00	£0.00	£5.00	£1.25	£0.00	£0.00
Voucher incentives	£0.00	£0.00	£0.00	£0.00	£130.00	£32.50	£0.00	£0.00
Refreshments[6]	£820.00	£74.55	£0.00	£0.00	£10.00	£.2.50	£0.00	£0.00
**Summary**								
Total cost	£6,842.13	£622.01	£957.33	£191.47	£1,013.90[7]	£253.48	£2,896.35	£724.09
Total cost per session	£131.58	£11.96	£119.67	£23.93	£202.78	£50.70	£103.44	£25.86

Note: 1 = Sessions were originally were 90 minutes, however when sandwiches were provided drug group facilitators arrived 30 minutes prior to session to be with participants while they ate, 2 = One-to-one EMDR sessions were 90 minutes and one-to-one Stabilisation sessions were 60 minutes, 3 = participants, 4 = Five participants were eligible for screening, however only four participants were successfully screened, 5 = Getting started groups were facilitated by two drug group facilitators, and 6 = Sandwiches and biscuits, 7 = Total cost without vouchers would have been £883.90 .

Heat map description: **£0.00** | **£0.01 - £99.99** | **£100 - £499.99** | **£500.00 - £1999.99** | **£2000.00 +**

For peer review only - http://bmjopen.bmj.com/site/about/guidelines.xhtml

Figure legend

Figure displaying the flow and numbers of potential and consented participants through the DUSSK study. Numbers may include more than one approach to potential participants by different recruiters. Includes reasons provided by women spoken to, but not screened, those screened but not eligible/ not consented and consented participants who did not reach the 'getting started' closed group intervention stage.

For peer review only

For peer review only - http://bmjopen.bmj.com/site/about/guidelines.xhtml

Figure 1: Flow of participants through the DUSSK study

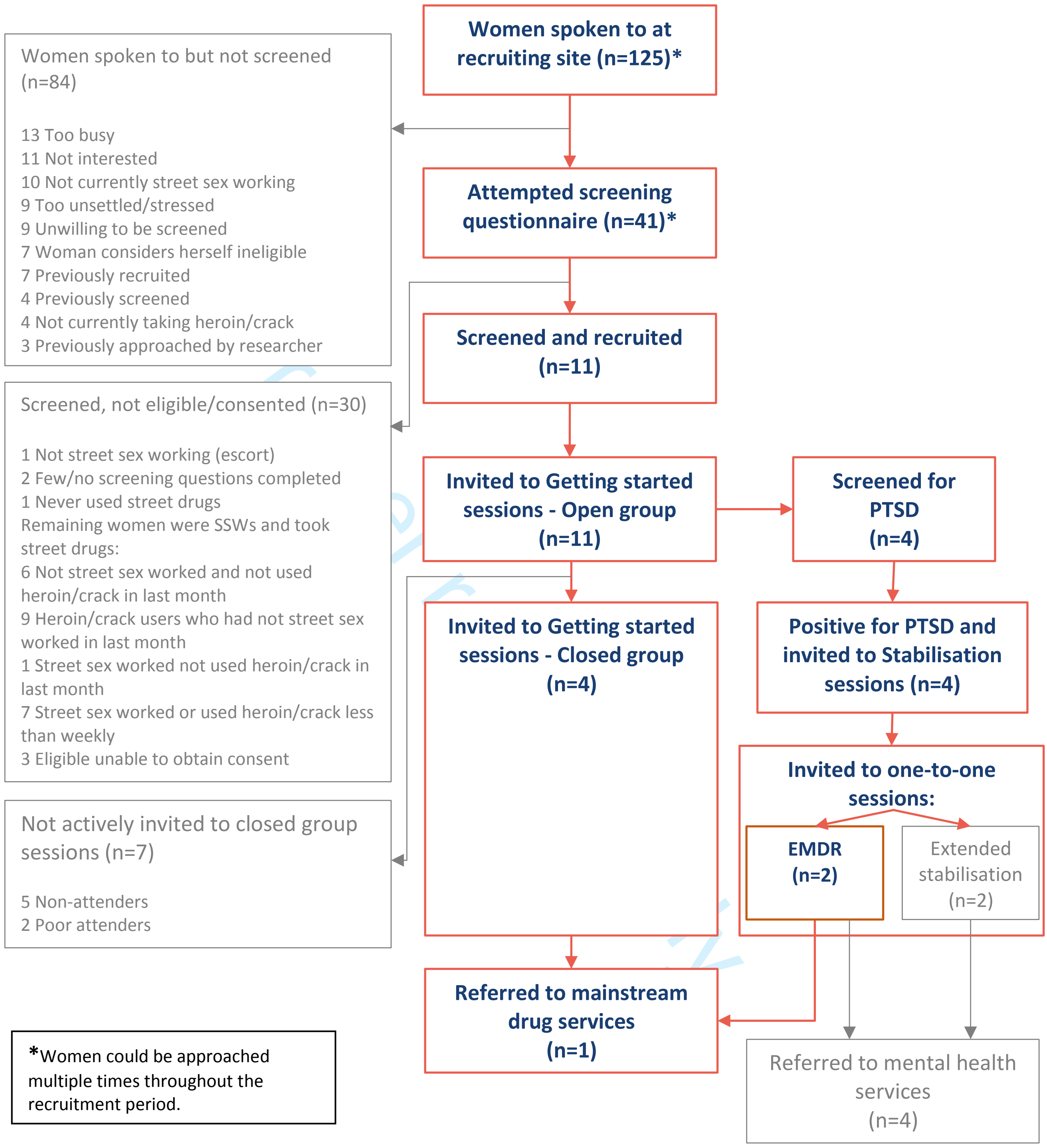

Reporting checklist for cohort study.

Based on the STROBE cohort guidelines.

Instructions to authors

Complete this checklist by entering the page numbers from your manuscript where readers will find each of the items listed below.

Your article may not currently address all the items on the checklist. Please modify your text to include the missing information. If you are certain that an item does not apply, please write "n/a" and provide a short explanation.

Upload your completed checklist as an extra file when you submit to a journal.

In your methods section, say that you used the STROBE cohortreporting guidelines, and cite them as:

von Elm E, Altman DG, Egger M, Pocock SJ, Gotzsche PC, Vandenbroucke JP. The Strengthening the Reporting of Observational Studies in Epidemiology (STROBE) Statement: guidelines for reporting observational studies.

		Reporting Item	Page Number
**Title and abstract**			
Title	#1a	Indicate the study's design with a commonly used term in the title or the abstract	1
Abstract	#1b	Provide in the abstract an informative and balanced summary of what was done and what was found	2
**Introduction**			
Background / rationale	#2	Explain the scientific background and rationale for the investigation being reported	4
Objectives	#3	State specific objectives, including any prespecified hypotheses	4
**Methods**			

For peer review only

Study design	#4	Present key elements of study design early in the paper	5
Setting	#5	Describe the setting, locations, and relevant dates, including periods of recruitment, exposure, follow-up, and data collection	5
Eligibility criteria	#6a	Give the eligibility criteria, and the sources and methods of selection of participants. Describe methods of follow-up.	5
Eligibility criteria	#6b	For matched studies, give matching criteria and number of exposed and unexposed	N/A
Variables	#7	Clearly define all outcomes, exposures, predictors, potential confounders, and effect modifiers. Give diagnostic criteria, if applicable	8
Data sources / measurement	#8	For each variable of interest give sources of data and details of methods of assessment (measurement). Describe comparability of assessment methods if there is more than one group. Give information separately for for exposed and unexposed groups if applicable.	7
Bias	#9	Describe any efforts to address potential sources of bias	N/A
Study size	#10	Explain how the study size was arrived at	7
Quantitative variables	#11	Explain how quantitative variables were handled in the analyses. If applicable, describe which groupings were chosen, and why	7
Statistical methods	#12a	Describe all statistical methods, including those used to control for confounding	8
Statistical methods	#12b	Describe any methods used to examine subgroups and interactions	N/A
Statistical methods	#12c	Explain how missing data were addressed	N/A
Statistical methods	#12d	If applicable, explain how loss to follow-up was addressed	N/A
Statistical methods	#12e	Describe any sensitivity analyses	N/A

For peer review only

Results

Participants	#13a	Report numbers of individuals at each stage of study—eg numbers potentially eligible, examined for eligibility, confirmed eligible, included in the study, completing follow-up, and analysed. Give information separately for for exposed and unexposed groups if applicable.	20
Participants	#13b	Give reasons for non-participation at each stage	9
Participants	#13c	Consider use of a flow diagram	20
Descriptive data	#14a	Give characteristics of study participants (eg demographic, clinical, social) and information on exposures and potential confounders. Give information separately for exposed and unexposed groups if applicable.	9
Descriptive data	#14b	Indicate number of participants with missing data for each variable of interest	21
Descriptive data	#14c	Summarise follow-up time (eg, average and total amount)	26
Outcome data	#15	Report numbers of outcome events or summary measures over time. Give information separately for exposed and unexposed groups if applicable.	9
Main results	#16a	Give unadjusted estimates and, if applicable, confounder-adjusted estimates and their precision (eg, 95% confidence interval). Make clear which confounders were adjusted for and why they were included	9-12
Main results	#16b	Report category boundaries when continuous variables were categorized	N/A
Main results	#16c	If relevant, consider translating estimates of relative risk into absolute risk for a meaningful time period	N/A
Other analyses	#17	Report other analyses done—e.g., analyses of subgroups and interactions, and sensitivity analyses	9-12

Discussion

Key results	#18	Summarise key results with reference to study objectives	13

For peer review only

Limitations	#19	Discuss limitations of the study, taking into account sources of potential bias or imprecision. Discuss both direction and magnitude of any potential bias.	13
Interpretation	#20	Give a cautious overall interpretation considering objectives, limitations, multiplicity of analyses, results from similar studies, and other relevant evidence.	15
Generalisability	#21	Discuss the generalisability (external validity) of the study results	N/A
**Other Information**			
Funding	#22	Give the source of funding and the role of the funders for the present study and, if applicable, for the original study on which the present article is based	16

The STROBE checklist is distributed under the terms of the Creative Commons Attribution License CC-BY. This checklist was completed on 16. December 2019 using https://www.goodreports.org/, a tool made by the EQUATOR Network in collaboration with Penelope.ai

For peer review only

BMJ Open

Drug Use in Street Sex worKers (DUSSK) study – results of a mixed methods feasibility study of a complex intervention to reduce illicit drug use in drug dependent female sex workers

Journal:	*BMJ Open*
Manuscript ID	bmjopen-2019-036491.R2
Article Type:	Original research
Date Submitted by the Author:	13-Oct-2020
Complete List of Authors:	Patel, Rita; University Hospitals Bristol NHS Foundation Trust, NIHR ARC West; University of Bristol, Population Health Sciences<br>Redmond, Niamh; University Hospitals Bristol NHS Foundation Trust, NIHR ARC West; University of Bristol, Population Health Sciences<br>Kesten, Joanna; University Hospitals Bristol NHS Foundation Trust, NIHR ARC West; University of Bristol, Population Health Sciences & NIHR Health Protection Research Unit in Evaluation of Interventions<br>Linton, Myles-Jay; University Hospitals Bristol NHS Foundation Trust, NIHR ARC West; University of Bristol, Population Health Sciences & Health Economics Bristol<br>Horwood, Jeremy; University Hospitals Bristol NHS Foundation Trust, NIHR ARC West; University of Bristol, Population Health Sciences & Centre for Academic Primary Care<br>Wilcox, David ; Avon and Wiltshire Mental Health Partnership NHS Trust, Acer Unit, Blackberry Hill Hospital, Manor Road,<br>Munafo , Jess; Avon and Wiltshire Mental Health Partnership NHS Trust, Acer Unit, Blackberry Hill Hospital, Manor Road,<br>Coast, Joanna; University Hospitals Bristol NHS Foundation Trust, NIHR ARC West; University of Bristol, Population Health Sciences & Health Economics Bristol<br>Macleod, John; University Hospitals Bristol NHS Foundation Trust, NIHR ARC West; University of Bristol, Population Health Sciences & Centre for Academic Primary Care<br>Jeal, Nicola; University Hospitals Bristol NHS Foundation Trust, NIHR ARC West; University of Bristol, Population Health Sciences
**Primary Subject Heading**:	Addiction
Secondary Subject Heading:	Health services research, Mental health, Public health, Health economics
Keywords:	QUALITATIVE RESEARCH, MENTAL HEALTH, HEALTH ECONOMICS, TRAUMA MANAGEMENT, Substance misuse < PSYCHIATRY

SCHOLARONE™
Manuscripts

I, the Submitting Author has the right to grant and does grant on behalf of all authors of the Work (as defined in the below author licence), an exclusive licence and/or a non-exclusive licence for contributions from authors who are: i) UK Crown employees; ii) where BMJ has agreed a CC-BY licence shall apply, and/or iii) in accordance with the terms applicable for US Federal Government officers or employees acting as part of their official duties; on a worldwide, perpetual, irrevocable, royalty-free basis to BMJ Publishing Group Ltd ("BMJ") its licensees and where the relevant Journal is co-owned by BMJ to the co-owners of the Journal, to publish the Work in this journal and any other BMJ products and to exploit all rights, as set out in our licence.

The Submitting Author accepts and understands that any supply made under these terms is made by BMJ to the Submitting Author unless you are acting as an employee on behalf of your employer or a postgraduate student of an affiliated institution which is paying any applicable article publishing charge ("APC") for Open Access articles. Where the Submitting Author wishes to make the Work available on an Open Access basis (and intends to pay the relevant APC), the terms of reuse of such Open Access shall be governed by a Creative Commons licence – details of these licences and which Creative Commons licence will apply to this Work are set out in our licence referred to above.

Other than as permitted in any relevant BMJ Author's Self Archiving Policies, I confirm this Work has not been accepted for publication elsewhere, is not being considered for publication elsewhere and does not duplicate material already published. I confirm all authors consent to publication of this Work and authorise the granting of this licence.

For peer review only

Drug Use in Street Sex worKers (DUSSK) study – results of a mixed methods feasibility study of a complex intervention to reduce illicit drug use in drug dependent female sex workers

Authors:

Dr Rita Patel*,[1,2] Dr Niamh M Redmond*,[1,2] Dr Joanna M Kesten,[1,2,3] Dr Myles-Jay Linton, [1,2,4] Dr Jeremy Horwood,[1,2,6] David Wilcox,[5] Jess Munafo,[5] Prof Joanna Coast,[1,2,4] Prof John Macleod,[1,2,6] Dr Nicola Jeal.[1,2,7]

*Joint first authors.

Author affiliations:

1 The National Institute for Health Research Applied Research Collaboration West (NIHR ARC West) at University Hospitals Bristol NHS Foundation Trust, Whitefriars, Lewins Mead, Bristol, UK, BS1 2NT

2 Population Health Sciences, Bristol Medical School, University of Bristol, Canynge Hall, 39 Whatley Road, Bristol, UK, BS8 2PS

3 The National Institute for Health Research Health Protection Research Unit in Evaluation of Interventions, University of Bristol, UK

4 Health Economics Bristol, Population Health Sciences, Bristol Medical School, University of Bristol, 1-5 Whiteladies Road, Bristol, UK, BS8 1NU

5 Avon & Wiltshire Partnership NHS Trust, Acer Unit, Blackberry Hill Hospital, Manor Road, Bristol, UK, BS16 2EW

6 Centre for Academic Primary Care, Population Health Sciences, Bristol Medical School, University of Bristol, Canynge Hall, 39 Whatley Road, Bristol BS8 2PS

7 Northern Devon Healthcare NHS Trust, Devon Sexual Health - North Devon, Barnstaple Health Centre, Vicarage Road, Barnstaple EX32 7BH

Corresponding author:

Dr Nicola Jeal (Nikki.Jeal1@nhs.net)

Word count:

Target Journal BMJ Open: Abstract: (304/300), Main: (4320/4000)

For peer review only

Abstract

Objectives

The majority of female street-based sex workers (SSWs) are dependent on illicit drugs and sell sex to fund their drug use. They typically face multiple traumatic experiences, starting at a young age, which continue through sex work involvement. Their trauma-related symptoms tend to increase when drug use is reduced, hindering sustained reduction. Providing specialist trauma care to address post-traumatic stress disorder (PTSD) alongside drug treatment may therefore improve treatment outcomes. Aims to (i) evaluate recruitment and retention of participants; (ii) examine intervention experiences and acceptability; and (iii) explore intervention costs using mixed methods feasibility study.

Setting

Female SSW charity premises in a large UK inner city.

Participants

Females aged 18 years or older, who have sold sex on the street and used heroin and/or crack cocaine at least once a week in the last calendar month.

Intervention

Female SSW-only drug treatment groups in a female SSW-only setting delivered by female staff. Targeted post-traumatic stress disorder (PTSD) screening then treatment of positive diagnoses with eye movement desensitization and reprocessing (EMDR) therapy by female staff from a specialist NHS trauma service.

Results

(i) Of 125 contacts 11 met inclusion criteria and provided informed consent, 4 reached the intervention final stage (ii) Service providers said working in collaboration with other services was valuable, the intervention was worthwhile and had a positive influence on participants. Participants viewed recruitment as acceptable and experienced the intervention positively. The unsettled nature of participant's lives was a key attendance barrier. (iii) The total cost of the intervention was £11,710, with staff costs dominating.

For peer review only

Conclusions

Recruitment and retention rates reflected study inclusion criteria targeting women with the most complex needs. Two participants received EMDR demonstrating that the three agencies working together was feasible. Staff heavy costs highlight the importance of supporting participant attendance to minimise per participant costs in a future trial.

Ethics approval

South West - Frenchay Research Ethics Committee (REC reference: 17/SW/0033; IRAS project ID: 220631). UK HRA approval was on 03/04/2017.The University of Bristol Research Enterprise and Development department sponsored this study (RED reference: RG2756).

Strengths and limitations of this study

- The novel intervention integrates a trauma-focussed treatment approach in order to reduce drug use in a challenging drug treatment population
- The intervention was delivered by specialists, reflecting the skills and experience required to appropriately manage the complex needs of the study population
- PPI formed an important part of the study methodology and informed each stage
- Recruitment took place over several months within an agency trusted and used daily by the study population to allow familiarisation with the researchers and multiple opportunities to participate
- This feasibility study design and methodology were not able to examine intervention effectiveness or cost effectiveness

Keywords:

sex workers, feasibility study, qualitative research, cost analysis, trauma treatment, drug dependency

For peer review only

Introduction

Most female street-based sex workers (SSWs) in the UK use heroin and/or crack cocaine.[1-3] Their drug dependency keeps them entrenched in a "work-score-use cycle",[4 5] which contributes to the morbidity and social instability typically seen in this group.[6]

Despite their drug treatment needs, drug dependent SSWs have poorer outcomes from drug treatment services compared to other service users,[7 8] sometimes due to stigma associated with their street sex work.[9] Previous SSW-focussed interventions aiming to reduce drug use have used educational,[10 11] substitute prescribing-based[12 13] and psychological[14] approaches but none robustly demonstrated a positive effect in reducing drug use.[15]

Poor mental health is a significant problem among SSWs.[16-18] Many have experienced multiple adversities in early life and during their involvement in sex work,[5 16 19] which exposes women to further risk of significant trauma.[16 17] Consequently, many SSWs are affected by post-traumatic stress disorder (PTSD).[16 17] Trauma symptoms, which often recur when drug use is reduced, may motivate a return to drug use.[20] Individual trauma-focused therapy alongside drug treatment may provide the best outcomes for reductions in drug use.[21-23] However to date, there is no robust evidence to demonstrate the impact of an integrated trauma-focussed treatment approach in reducing drug use amongst female drug dependent SSWs.

In collaboration with SSWs and service providers, and informed by existing research[9 15]we developed a novel intervention, to simultaneously address the unique and complex combination of drug use and PTSD in female drug-dependent SSWs. The intervention proposes an integrated care pathway through an innovative multi-agency partnership.[24] We report here the results of the DUSSK (Drug Use in Street Sex worKers) feasibility study, which aimed to (i) evaluate the recruitment and retention of SSWs to the intervention; (ii) examine the experience and acceptability of the intervention for participants and service providers; and (iii) explore costs to service providers associated with the intervention.

For peer review only - http://bmjopen.bmj.com/site/about/guidelines.xhtml

Methods

Study design, setting and eligibility

Detailed methods are described in the published protocol.[24] This mixed methods feasibility study took place in a UK inner city setting. Females aged 18 years or older, who sold sex on the street at least weekly in the last calendar month and used heroin and/or crack cocaine at least once a week in the last calendar month, were eligible to participate.[24] The intervention was delivered at SSW charity premises, which supplied support, health and advocacy services.[24]

Recruitment

The recruitment target for this feasibility study was 30 women.[24] Local organisations that SSWs were known to access were provided with study promotional materials. One of three researchers (JMK, NJ, SR) attended an SSW support and advocacy charity, at least twice a week, to directly approach potential participants; alternatively, interested SSWs could telephone researchers. This approach meant that women were potentially approached and counted as contacts several times during recruitment. Eligibility screening was conducted face-to-face or via telephone. Women gave fully informed, written consent to participate in the study and baseline data were also collected at the time of recruitment. To maintain safety and confidentiality, each participant provided details of acceptable ways in which to be contacted. Screening data were retained and remained anonymised for those not recruited.

Patient and Public Involvement (PPI)

Women with experience of street sex work and drug-dependency took part in focus groups and one-to-one discussions with NJ to inform study design, processes, documentation and intervention development. On commencement of the study, a group of women who were ineligible for recruitment were approached for involvement in PPI. They addressed challenges with recruitment, participation and adherence issues (described below) and suggested solutions, which were implemented. For example, they recommended changes such as provision of sandwiches to improve attendance.

For peer review only - http://bmjopen.bmj.com/site/about/guidelines.xhtml

The intervention

The intervention consisted of SSW only drug treatment groups, targeted screening for PTSD symptoms (one-to-one clinical interview and PTSD checklist- PCL5)[25] and, if positively diagnosed, one-to-one EMDR therapy, all delivered by female staff through a collaboration between three service providers (National Health Service (NHS) trauma services, the SSW and drug treatment charities). The intervention was designed so participants were initially invited to attend a weekly 'Getting started' drug treatment group to reduce fear and anxiety about engaging in a group setting and get used to the format and level of disclosure expected. Participants were to progress to a 'Preparation for recovery' drug treatment group which focused on people's barriers to motivation for change, examining pros and cons of drug use and exploring triggers for using drugs to enable participants to achieve a level of drug use stability. As stated in the protocol[24] and in line with provider's usual care, participants were perceived as demonstrating drug use stability by exhibiting evidence of life/drug use stability such as engagement and functioning in the group, positive interaction with group facilitators and regular opioid substitution therapy (OST) by the group facilitators. When group facilitators judged participants were achieving drug use stabilisation, and they had attended three sessions consecutively, they were offered screening for PTSD symptoms by a female clinical psychologist. Those experiencing PTSD symptoms were invited to attend five PTSD 'Stabilisation' group sessions, facilitated by the same female clinical psychologist, to equip participants with the skills to self-soothe and reorientate in preparation for the one-to-one EMDR treatment. Once all 'Stabilisation' group sessions had been completed, the clinical psychologist assessed participants for readiness for one-to-one EMDR sessions and if eligible, participants progressed to a course of 12 sessions with the clinical psychologist on a weekly, or fortnightly, basis. Trauma treatment (PTSD screening, stabilisation groups and one-to-one) ran in parallel to the drug treatment groups, Figure 1 (red boxes) shows the planned flow of participants through the study.

The intervention proceeded as described in the protocol paper [24] with the following changes (bulleted below):

- Participants were encouraged to attend all sessions with the offer of car lifts, bus tickets and taxis, in addition to the planned weekly phone calls and texts, by the staff at partner agencies.

'Getting started' and 'Preparation for recovery' drug treatment groups

- Retendering resulted in a change of drug group service provider which, along with low numbers recruited, resulted in the drug groups merging into a single open drug treatment 'Getting started' group.

For peer review only - http://bmjopen.bmj.com/site/about/guidelines.xhtml

- Attendance at three consecutive sessions was required to move onto the next group, instead of attendance at any four.
- Sandwiches were supplied prior to the single drug group to support attendance from the 14th session onwards.
- The number of sessions continued beyond those initially planned due to delays in PTSD screening (see below).
- Poor attendance, the participant's unstable behaviour and the intervention running for longer than planned (due to the retendering of drug services) affected the drug group facilitators' ability to deliver structured content. An art worker was included in 4 sessions to maintain participant's interest and engagement with sessions.
- PTSD screening was halted from the 29th session onwards, due to the limited remaining study time to complete the intervention, meaning the drug group became closed and only included PTSD screened participants.

Screening for PTSD

- Delays occurred to both the organisation of the PTSD screening and PTSD stabilisation group set up due to lack of capacity and delays in NHS trauma service staff recruitment. Therefore, some participants had long gaps between recruitment and PTSD screening and subsequent stabilisation group sessions.

PTSD 'Stabilisation' group

- Incentives of £10 vouchers per session were offered for attendance (mandatory sessions).

One-to-one EMDR therapy for PTSD

- Some sessions were scheduled in a private rented room (due to availability issues) in a local community centre and not at the SSW charity premises.
- Two participants were offered weekly one-to-one extended stabilisation sessions (8 maximum) with the clinical psychologist rather than EMDR therapy as this was deemed the most appropriate treatment.

For peer review only - http://bmjopen.bmj.com/site/about/guidelines.xhtml

Data collection methods

Sample size and quantitative data collection

A formal sample size calculation was not conducted as the aim was to assess feasibility.[24] Self-reported levels of illicit drug use, involvement in SSW, completion of PTSD Checklist PCL5[25] and demographics were collected at the time of consent. Attendance registers were taken at the start of each group or one-to-one session by the facilitator(s).

Qualitative data collection

With participants' verbal consent one non-participant observation of a drug group was conducted to understand delivery, examine interactions and intervention experiences, with brief notes taken during the group.[26]

In-depth semi-structured interviews were conducted with participants and service providers either face-to-face or by telephone. Participants were interviewed after intervention completion or study drop out. Consent to contact participants regarding interviews was sought at recruitment. Additional written or audio recorded verbal, informed consent was obtained prior to all interviews. Interviews explored views and experiences of the intervention and how to improve acceptability. Participants received a £20 high street shopping voucher for taking part. Most service provider interviews were conducted at the end of the intervention period and also sought to understand operational issues, inter-agency working and intervention delivery.

Economic data collection

Resource use information (2018 £GBP) was collected prospectively by the agencies. Data collection focussed on four categories: Staff time, Facilities, Travel (provider funded staff and patient transport), and Materials.

Non-attendance was dealt with as follows; if no participants arrived after 45 minutes for a 'getting started' session, staff left and were only costed for the time that they spent waiting. Staff delivering 'trauma screening', '1-1s' and 'stabilisation groups' were costed for all sessions booked, regardless of non-attendances.

For peer review only - http://bmjopen.bmj.com/site/about/guidelines.xhtml

Data analysis

The integration of qualitative and quantitative data used the established 'following a thread' technique [27] where key themes were traced using all data sets.

Statistical analysis

Descriptive statistics (using Stata version14) were used to monitor recruitment and retention via CONSORT statement[28] style flowcharts, and to examine participant demographics, questionnaire responses and attendance.

Qualitative analysis

Interviews were conducted by JMK and NJ, and the audio files transcribed, anonymised and checked for accuracy. QSR NVivo 10 software was used to perform inductive thematic analysis[29] using constant comparison techniques.[30 31] A preliminary coding framework was developed by JMK and discussed with the multidisciplinary research team JH and NJ to ensure credibility and confirmability.

Cost analysis

Costs to the service provider were examined and summarised separately for each of the four intervention components. Staff costs were calculated using salaries and on-costs or generated using standard unit cost data available for health and social care professionals.[32] Facility costs were calculated based on similar space rental options. Total cost, total cost per eligible participant, total cost per session held, and total cost per session per eligible participant were calculated for each intervention component. Cost data were tabulated using principles of heat-map methodology, where colour lightness is used to communicate the magnitude of different costs.[33 34]

For peer review only - http://bmjopen.bmj.com/site/about/guidelines.xhtml

Results

Recruitment was from November 2017 to March 2018 with the intervention delivered until December 2018.

(i) Recruitment and retention

Recruitment Process

Approximately 400 flyers and nine posters were distributed. Potential participants were spoken to by three researchers at 37 three-hour 'drop-in' sessions at the SSW charity. Of 125 contacts made with women, 84 declined screening. Reasons for decline included being too busy (n=13), not interested (n=11) or reporting not currently street sex working (n=10). Fourteen approaches were reported as repeat approaches, with seven reporting previous recruitment and four previous screening (as the researchers recruited on different weekdays and screening data was anonymous). Figure 1 details the flow of screened and recruited participants through the intervention.

Of 41 women screened, 11 were eligible and consented to participate, three were eligible but unable to give consent (two were too distressed and one had health issues preventing participation). Of the 27 ineligible women, two did not fully complete the screening questions. The main reasons for exclusion related to ineligible frequency of drug use and/or sex work. Table 1 shows the range of days since responders last street sex worked or had taken heroin/crack by those recruited and not recruited.

Of 11 participants consenting to be invited for qualitative interviews, five were uncontactable. Seven interviews were conducted with six participants (six face-to-face and one via telephone); four interviewed participants received all components of the intervention. Ten service provider interviews were conducted with representatives from the drug treatment service (n=4), the trauma service (n=2), and the SSW charity (n=4). Table 2 details quotes from the interviews to support the main results described below.

Acceptability of recruitment

The recruitment process was described as acceptable by most participants and service providers. Face-to-face recruitment was experienced as confidential, with participants reporting receiving clear

For peer review only - http://bmjopen.bmj.com/site/about/guidelines.xhtml

explanations of the study. Recruitment over five months within the SSW charity offered multiple opportunities to participate and was acceptable. Most service providers interviewed reflected that if study inclusion criteria were broadened to include less regular drug use, participants with more lifestyle stability could have been recruited, and whom may have found the intervention easier to engage with. However, some felt that creating groups with diverse levels of drug use could have negative consequences for example, risk of relapse for those who had reduced their drug use.

Group attendance

All 11 consented participants were invited to attend drug treatment groups. However, participants attendance varied throughout the study, with participants sometimes arriving late or leaving early (Table 3). Four attended 30-76% of sessions compared to 7 attenders who attended only 0-18% of sessions. The five most frequent attenders were invited to PTSD screening of which four attended and were all found to have symptoms of PTSD. All four PTSD screened participants attended 20-100% of the stabilisation groups with the clinical psychologist. At the end of the stabilisation groups, two participants were deemed suitable for EMDR therapy by the clinical psychologist and two were offered extended stabilisation sessions. All four participants attended some one-to-one sessions (Table 3) but missed at least two consecutive trauma treatment appointments with the clinical psychologist and had to withdraw from the sessions. However, all participants were referred to further mental health services and one participant was also referred to mainstream drug services.

Facilitators to attendance

Service providers across all partner agencies sent reminders to participants, which were described as helpful and appreciated by participants. One SSW charity service provider played a vital role in encouraging attendance through reminding participants to attend, arranging transport (taxi, bus or driving participants to sessions) and helping participants prepare for the intervention. Provision of sandwich lunches before the groups was seen by service providers and participants as helpful for encouraging attendance, facilitating a relaxed start to groups and supporting concentration. Vouchers were also viewed as encouraging attendance by participants and service providers.

Barriers to attendance

The unsettled nature of participant's lives was perceived as an attendance barrier and was underpinned by problematic drug use, poor adherence to OST, sex work, tiredness and poor mental health. Arguments between participants, a lack of readiness to confront issues with drugs and trauma

For peer review only - http://bmjopen.bmj.com/site/about/guidelines.xhtml

and an absence of social support were described as making attendance difficult. Delays to screening referral and trauma treatment were also reported to negatively affect participants' motivation.

(ii) Experience and acceptability of the intervention

Initial impressions

All participants perceived the intervention as valuable and welcome. Common reasons given by participants for taking part included the opportunity for 'change', greater stability and valuing the opportunity to combine mental health and drug treatment. Service providers viewed the intervention as a novel opportunity for 1) SSWs to receive mental health treatment while continuing to use drugs and 2) to address the barriers to mainstream drug treatment. Some service providers highlighted the challenge for participants to process trauma while continuing to use drugs and potential risks of sex working when receiving trauma treatment.

Service providers' views on the intervention

The drug group facilitators described enjoying delivering the groups and building good relationships with participants. However, they described some drug sessions as intense and difficult to manage due to participants' distress, accounts of trauma and chaotic behaviour. The need for appropriate support and supervision of facilitators was highlighted as a requirement to manage these challenges.

A clinical psychologist suggested that without the 're-traumatising' effects of street sex work, the effectiveness of the trauma processing in the trauma treatment might be enhanced. Service providers also proposed extending the stabilisation work to develop the effectiveness of the trauma treatment and recommended the intervention offer alternatives to EMDR to suit individual participants' needs.

Service providers said working in partnership with other specialist services to deliver the intervention was valuable, there was mutual respect and good communication between staff. It was suggested it would have been useful to have collaborative, regular case-review meetings between the services to assess the progress and needs of the participants and enhance the communication channels.

Participants views on the intervention

Participants described generally positive intervention experiences. They described forming meaningful relationships with the drug group facilitators and clinical psychologist. They liked that the

For peer review only - http://bmjopen.bmj.com/site/about/guidelines.xhtml

groups were female and sex worker-only, they knew other participants already and could speak openly about, and relate to, one another's experience of trauma, drug use and street sex work. Participants also valued that the intervention was delivered at SSW charity premises, which was liked for its familiarity, safety, comfort, convenience and freedom from judgement and shame. The day of the week, time and frequency of sessions, drug group session length and group size were also acceptable to most participants and service providers. These factors overcame some of the barriers participants highlighted to attending mainstream drug services.

Strengths of the intervention

Through the intervention, participants reflected on their need to address their trauma and drug use. Some acknowledged that they were not ready to address their trauma but aspired to this in future, having had positive experiences of therapy during the intervention. Participants attributed improved wellbeing, coping strategies and perceptions of self-worth to the intervention. One participant was seen less on the SSW outreach van (an indicator of sex working) and, significantly, stopped using her working name, signifying 'taking back ownership of who she is'. One drug group facilitator felt the flexibility of the study intervention was able accommodate participants' unstable lives and level of trauma that would have prevented them from complying with the rules of conduct in mainstream drug services and thus prevented them from receiving treatment to address their needs. They also felt that an additional positive feature of the intervention was participants being able to discuss their sex work, due to the female SSW only membership of the groups.

Participation in the intervention was described by SSW charity service providers and two participants as supporting and empowering the participants to engage with clinical and support services to address their needs. Another participant felt she used the SSW charity services less now because she needed less support.

(iii) Cost analysis

The total cost of the intervention was £11,710, with staff costs being the largest component (Table 4). The most expensive component of the intervention was the 'Getting started' sessions (which totalled £6,842). Despite having the second to lowest sub-total cost across the intervention (£1,014), the stabilisation groups had the highest cost per session held (£203). Although the one-to-one sessions had the lowest cost per session held (£103), the larger number of sessions at this point resulted in this section having the highest cost per eligible participant (n=4, £724). Trauma screening had the lowest cost per eligible participant (n=5, £191)

For peer review only - http://bmjopen.bmj.com/site/about/guidelines.xhtml

Fidelity

The intervention was broadly delivered as intended incorporating suggested planned changes to the protocol, it was more flexible and less linear than originally planned.[24] Delays in PTSD screening meant that there was only a single drug group, which continued for longer than originally planned.

Discussion

Summary of findings

This study used a mixed methods approach to investigate the feasibility and acceptability of a novel, complex intervention to reduce illicit drug use in female drug-dependent SSWs. We demonstrated that drug-dependent SSWs could maintain attendance at female SSW-only drug group sessions and the integrated trauma-focussed treatment approach, in a trusted and supportive environment, with intensive support from SSW charity staff. Recruitment was lower than anticipated, with four participants PTSD screened and whom met criteria for PTSD. They progressed through to the final stage of the intervention, all four participants were ultimately able to access mental health services and one began the process of accessing mainstream drug services. Participants and service providers mostly experienced the recruitment process, the intervention and delivery mechanisms (especially the SSW-only environment) positively. Managing SSW trauma disclosure proved challenging for drug group facilitators and non-clinical staff and resulted in the recommendation that there is additional training and support for staff in future studies. The need for intervention refinement, for example, extending drug stabilisation sessions, were suggested to provide additional support prior to trauma treatment. Attendance and adherence barriers primarily related to the issues the intervention sought to address, namely problematic drug use, sex work, and poor mental health, rather than the acceptability of the intervention itself. The total cost of the intervention was £11,710, with staff costs dominating.

Strengths and limitations of this study

Strengths of our study were that recruiting researchers also conducted the interviews, which may have facilitated a rapport with participants and supported more open and honest reflections. Secondly, all aspects of the intervention were delivered by specialists which was necessary for this high-risk group with multiple complex co-morbidities. Thirdly, PPI formed an important part of the study and informed each stage. Fourth, recruitment over several months with multiple approaches

For peer review only - http://bmjopen.bmj.com/site/about/guidelines.xhtml

allowed SSWs many opportunities to take part. This approach took account of the rapidly changing lives of SSWs resulting in changing eligibility status as well as allowing time for them to become familiar with the researchers. Finally, recruitment within a trusted agency may have had a positive influence on recruitment.

This feasibility study provides only preliminary information on the intervention performance and cost and does not examine the effectiveness or potential for reducing costs in other parts of the health service or wider society. Delays due to changes to service provision are likely to have adversely influenced study recruitment and retention, with delays resulting in higher service costs, but reflect the real-life issues facing multi-agency work.

Comparison with other research

This feasibility study is the first, to our knowledge, to address previously highlighted barriers to effective drug treatment for SSWs.[15 22 23] Through incorporating female SSW-only drug groups alongside an intervention with specialised trauma treatment, delivered in a female SSW-only setting by female staff[22 24] we showed how an integrated treatment approach in this complex vulnerable group could feasibly be implemented and delivered, with changes to the intervention, albeit at a higher than expected cost, mostly due to the delays incurred due to service retendering.

This study is the first interventional study to employ clinical staff from a specialist trauma service to deliver EMDR to address trauma symptoms as part of the drug treatment process for SSWs. These women have been found to have high levels of poor mental health,[16 18] particularly trauma,[5 16 17] which contributes to poor drug treatment outcomes.[7 8] The intervention took account of SSWs frequent experience of abuse and violence,[35 36] and recommendations for female-only trauma-focussed drug treatment interventions[22 37 38] for treatment of PTSD and long term drug use reduction. Some of the characteristics the intervention sought to address presented as barriers to attendance and retention, however these are common in studies trying to effect behaviour changes within vulnerable groups.[23 39]

Previous SSW-focussed interventions aiming to reduce drug use[10-13 14] were unable to demonstrate strong evidence of a positive effect[15] suggesting the need for a novel approach with evidence of efficacy assessed through a robust methodological approach. The highlighted barriers to attendance, engagement and delivery of the intervention are in keeping with other studies[15 23 39] but indicate that further changes to the DUSSK feasibility study design are likely to be required in future studies.

For peer review only - http://bmjopen.bmj.com/site/about/guidelines.xhtml

Conclusion and implications for service provision and research

This study sought to explore the feasibility of delivering a novel complex intervention to a very challenging population with high levels of unmet need. Inclusion criteria were informed by PPI, clinical expertise and academic literature. They targeted women who were likely to benefit the most from a trauma-based intervention but whose drug dependency and chaotic lives made them challenging study participants to recruit and retain. However, all four of the participants screened for PTSD were diagnosed, revealing the unmet need for trauma treatment. Though unsurprising, the severity of trauma disclosed by SSWs proved unexpectedly challenging for service providers.[22 37 38] Further data to understand the extent and severity of PTSD in SSWs is recommended to inform service provision. Overall, the experiences described by those receiving the intervention, suggest it is an acceptable approach to reducing SSWs drug use.

The three services found the intervention valuable and were able to work together effectively despite setbacks such as changing contracts and service pressures. They also suggested more staff support for managing trauma disclosure, extended stabilisation sessions and closer working could improve intervention delivery.

Intervention costs were driven up by poor participant attendance, though staff pressures and the retendering process increased the length (and cost) of the intervention period. However, decreased SSW use of health services, the criminal justice system and impacts of criminal activity on wider society may justify its adoption if future trials demonstrate intervention effectiveness in reducing drug use.

In order to support future interventional trials in this important field where there are few effectiveness studies we recommend the following study refinements for consideration.

1. The intervention could also include women with more stability in their lives to increase recruitment and retention.
2. Regular meetings throughout the study enabling all service providers involved in intervention delivery to express concerns and seek to understand participants needs from the perspective of different professionals so there is effective multi-agency support for individual participants where needed.
3. Training for all involved staff in managing the disclosure of trauma.
4. Support and encouragement for participant engagement through provision of transport to and refreshments prior to treatment sessions

For peer review only - http://bmjopen.bmj.com/site/about/guidelines.xhtml

5. Intervention flexibility and responsiveness in offering trauma-focussed alternatives to EMDR which may be more suitable for individual participants needs.
6. An extended trauma therapy programme, including extended stabilisation therapy prior to trauma treatment, to accommodate the complexity of SSW needs.

For peer review only

For peer review only - http://bmjopen.bmj.com/site/about/guidelines.xhtml

Additional information

Acknowledgements

The authors are extremely grateful to all the women who have participated in the study; all service providers and all other staff whose participation made this study possible. They would like to thank the PPI group for their time, thoughts and suggestions. We are grateful to the following organisations and individuals who have helped with the study for their time, expertise and support: Developing Health and Independence, Bristol Drugs Project, Avon & Wiltshire Mental Health Partnership NHS Trust and One25 charity, Katie Warner, Lucy Pettler, Rosie Davies, Maggie Telfer, Elinor Griffiths, Gill Nowland, Sophie Ramsden, Elaine Driver, Anna Smith, Rhea Warner, Jennifer Riley, Tracey Tudor, Madeline Saunders, Charlotte Hignell, Sophie Banks, Jane Bowman, Sarah Shatwell, Katrina Turner, Hasina Downie, Jo Daniels and Louisa Chowen.

Author Contributions

NJ, NMR, JH, JMK, RP, DW, JM, JMa, MJL and JC are responsible for the study design, collection of data and analysis. NMR, RP, NJ and JH are responsible for study management and coordination. JMK and NJ conducted the interviews. JMK led the qualitative analysis in collaboration with NJ and JH. RP conducted the quantitative analysis. MJL, RP and JC conducted the costing study. NMR, RP, JMK, MJL, JC, NJ and JH drafted the paper. All authors read, commented on and approved the final manuscript.

Funding

The research is supported by a National Institute for Health Research (NIHR) Clinic Trials Fellowship awarded to NJ (NIHR-CTF-2016-05-07), the National Institute for Health Research Collaboration for Leadership in Applied Health Research and Care West (NIHR CLAHRC West P324), now recommissioned as NIHR Applied Research Collaboration West (NIHR ARC West) and Research Capability Funding awarded by University Hospitals Bristol NHS Foundation Trust (RCF 2016-17-18). JMK is partly funded by NIHR Health Protection Research Unit in Evaluation of Interventions. The views expressed are those of the authors and not necessarily those of the NIHR or the Department of Health and Social Care.

Competing interests

None declared

For peer review only

Data availability statement

All data relevant to the study are included in the article.

For peer review only

For peer review only - http://bmjopen.bmj.com/site/about/guidelines.xhtml

References

1. Cusick L, Martin A, May T. Vulnerability and involvement in drug use and sex work. Home Office research study 268. London: Home Office, 2003.
2. Deering KN, Kerr T, Tyndall MW, et al. A peer-led mobile outreach program and increased utilization of detoxification and residential drug treatment among female sex workers who use drugs in a Canadian setting *Drug & Alcohol Dependence* 2011;113(1):46-54.
3. Morris D, Lemus H, Wagner K D, et al. Factors associated with pathways toward concurrent sex work and injection drug use among female sex workers who inject drugs in northern Mexico. *Addiction* 2013;108:161-70.
4. Jeal N, Salisbury C, Turner K. The multiplicity and interdependency of factors influencing the health of street-based sex workers: a qualitative study. *Sex Transm Infect* 2008;84(5):381-5. doi: 10.1136/sti.2008.030841 [published Online First: 2008/07/04]
5. Gorry J, Roen K, Reilly J. Selling your self? The psychological impact of street sex work and factors affecting support seeking. *Health Soc Care Community* 2010;18(5):492-9. doi: 10.1111/j.1365-2524.2010.00925.x [published Online First: 2010/06/30]
6. Jeal N, Salisbury C. Health needs and service use of parlour-based prostitutes compared with street-based prostitutes: a cross sectional survey. *BJOG* 2007;114:875-81.
7. Burnette M LE, Ilgen M, Frayne S, Mayo J, Weitlauf J. Prevalence and health correlates of prostitution among patients entering treatment for substance use disorders. *Archives of General Psychiatry* 2008;65(3):337 - 44.
8. Burnette M SR, Timko C, Ilgen M. Impact of substance-use disorder treatment on women involved in prostitution: substance use, mental health and prostitution one year after treatment. *Journal of Studies on Alcohol and Drugs* 2009;70:32-40.
9. Jeal N, Macleod J, Salisbury C, et al. Identifying possible reasons why female street sex workers have poor drug treatment outcomes: a qualitative study. *BMJ Open* 2017;7(3):e013018. doi: 10.1136/bmjopen-2016-013018 [published Online First: 2017/03/25]
10. Sherman SG, German D, Cheng Y, et al. The evaluation of the JEWEL project: An innovative economic enhancement and HIV prevention intervention study targeting drug using women involved in prostitution. *Aids Care-Psychological and Socio-Medical Aspects of Aids/Hiv* 2006;18(1):1-11. doi: 10.1080/09540120500101625
11. Surratt HL, Inciardi JA. An effective HIV risk-reduction protocol for drug-using female sex workers. *J Prev Interv Community* 2010;38(2):118-31. doi: 10.1080/10852351003640732 [published Online First: 2010/04/15]
12. Gunne LM, Gronbladh L, Petersson S. [Methadone treatment in the prevention of AIDS. Heroin-dependent prostitutes are an important target]. *Lakartidningen* 1986;83(49):4194-6. [published Online First: 1986/12/03]
13. Litchfield J, Maronge A, Rigg T, et al. Can a targeted GP-led clinic improve outcomes for street sex workers who use heroin? *The British journal of general practice : the journal of the Royal College of General Practitioners* 2010;60(576):514-6. doi: 10.3399/bjgp10X514774 [published Online First: 2010/07/03]
14. Yahne CE, Miller WR, Irvin-Vitela L, et al. Magdalena Pilot Project: motivational outreach to substance abusing women street sex workers. *J Subst Abuse Treat* 2002;23(1):49-53. [published Online First: 2002/07/20]
15. Jeal N, Macleod J, Turner K, et al. Systematic review of interventions to reduce illicit drug use in female drug-dependent street sex workers. *BMJ Open* 2015;5(11):e009238. doi: 10.1136/bmjopen-2015-009238 [published Online First: 2015/11/20]
16. El-Bassel N, Schilling R, Irwin K, et al. Sex trading and psychological distress among women recruited from the streets of Harlem. *American Journal Public Health* 1997;87:66-70.
17. Chukakov B, Ilan K, Belmaker RH, et al. The motivation and mental health of sex workers. *Journal of Sex & Marital Therapy* 2002;.28(4):pp. doi: 10.1080/00926230290001439

For peer review only - http://bmjopen.bmj.com/site/about/guidelines.xhtml

18. Roxburgh A, Degenhardt L, J C, et al. Drug Dependence and Associated Risks Among Female Street-Based Sex Workers in the Greater Sydney Area, Australia. *Substance Use & Misuse* 2008;43(8-9):1202-17.
19. Mellor R, Lovell A. The lived experience of UK street-based sex workers and the health consequences: an exploratory study. *Health promotion international* 2012;27(3):311-22. doi: 10.1093/heapro/dar040 [published Online First: 2011/07/06]
20. Reynolds M, Mezey G, Chapman M, et al. Co-morbid post-traumatic stress disorder in a substance misusing clinical population. *Drug and alcohol dependence* 2005;77(3):251-58. doi: https://doi.org/10.1016/j.drugalcdep.2004.08.017
21. Cloitre M, Courtois CA, Charuvastra A, et al. Treatment of complex PTSD: Results of the ISTSS expert clinician survey on best practices. *Journal of Traumatic Stress* 2011;24(6):615-27. doi: 10.1002/jts.20697
22. Roberts NP, Roberts PA, Jones N, et al. Psychological therapies for post-traumatic stress disorder and comorbid substance use disorder. *Cochrane Database Syst Rev* 2016;4(4):CD010204. doi: 10.1002/14651858.CD010204.pub2 [published Online First: 2016/04/05]
23. Bailey K, Trevillion K, Gilchrist G. What works for whom and why: A narrative systematic review of interventions for reducing post-traumatic stress disorder and problematic substance use among women with experiences of interpersonal violence. *Journal of Substance Abuse Treatment* 2019;99:88-103. doi: 10.1016/j.jsat.2018.12.007
24. Jeal N, Patel R, Redmond NM, et al. Drug use in street sex workers (DUSSK) study protocol: a feasibility and acceptability study of a complex intervention to reduce illicit drug use in drug-dependent female street sex workers. *BMJ Open* 2018;8(11):e022728. doi: 10.1136/bmjopen-2018-022728 [published Online First: 2018/11/06]
25. Bovin MJ, Marx BP, Weathers FW, et al. Psychometric properties of the PTSD Checklist for Diagnostic and Statistical Manual of Mental Disorders-Fifth Edition (PCL-5) in veterans. *Psychol Assess* 2016;28(11):1379-91. doi: 10.1037/pas0000254 [published Online First: 2016/11/04]
26. Emerson RM, Fretz RI, Shaw LL, et al. Writing ethnographic fieldnotes. Chicago and London: University of Chicago Press 1995.
27. Moran-Ellis J, Alexander VD, Cronin A, et al. Triangulation and integration: processes, claims and implications. 2006;6(1):45-59. doi: 10.1177/1468794106058870
28. Schulz KF, Altman DG, Moher D, et al. CONSORT 2010 statement: updated guidelines for reporting parallel group randomised trials. *BMJ* 2010;340:c332. doi: 10.1136/bmj.c332 [published Online First: 2010/03/25]
29. Braun V, Clarke V. Using thematic analysis in psychology. *Qualitative Research in Psychology* 2006;3(2):77-101.
30. Glaser B, Strauss A. The Constant Comparative method of Qualitative Analysis. In the Discovery of Grounded Theory: Strategies for Qualitative Research Chicago: Aldine 1967:101-158.
31. Charmaz K. Constructing grounded theory: a practical guide through qualitative analysis. London: Sage Publications, Inc 2006.
32. Curtis LB, A. . Unit Costs of Health and Social Care University of Kent, Canterbury: Personal Social Services Research Unit 2018.
33. Kirk A. Data Visualization: a successful design process: Packt Publishing Ltd 2012.
34. Iliinsky N, Steele J. Designing data visualizations: Representing informational Relationships: O'Reilly Media, Inc. 2011.
35. Ulibarri MD, Hiller SP, Lozada R, et al. Prevalence and characteristics of abuse experiences and depression symptoms among injection drug-using female sex workers in Mexico. *J Environ Public Health* 2013;2013:631479. doi: 10.1155/2013/631479 [published Online First: 2013/06/06]

For peer review only - http://bmjopen.bmj.com/site/about/guidelines.xhtml

36. Monica D. Ulibarri, Steffanie A. Strathdee, Remedios Lozada, et al. Prevalence and Correlates of Client-Perpetrated Abuse Among Female Sex Workers in Two Mexico–U.S. Border Cities. *Violence Against Women* 2014;20(4):427– 45.
37. Greenfield SF, Brooks AJ, Gordon SM, et al. Substance abuse treatment entry, retention, and outcome in women: a review of the literature. *Drug and alcohol dependence* 2007;86(1):1-21. doi: 10.1016/j.drugalcdep.2006.05.012 [published Online First: 2006/06/09]
38. Wiechelt SA, Shdaimah CS. Trauma and Substance Abuse Among Women in Prostitution: Implications for a Specialized Diversion Program. *Journal of Forensic Social Work* 2011;1(2):159-84. doi: 10.1080/1936928x.2011.598843
39. Bailey K, Trevillion K, Gilchrist G. “We have to put the fire out first before we start rebuilding the house”: practitioners’ experiences of supporting women with histories of substance use, interpersonal abuse and symptoms of post-traumatic stress disorder. *Addiction Research & Theory* 2019:1-9. doi: https://doi.org/10.1080/16066359.2019.1644323

For peer review only

Figure 1 Flow of participants through the DUSSK study

For peer review only

For peer review only - http://bmjopen.bmj.com/site/about/guidelines.xhtml

Table 1 Characteristics of screened women

	Screened not recruited (N=30)		Screened and recruited (N=11)	
	**N (%) ***	**Median (range)**	**N (%) ***	**Median (range)**
Female	27 (90)		11 (100)	
Age	23 (77)	37 (26-55)	11 (100)	38 (23-53)
Ever sold sex on the street?				
Yes	26 (87)		11 (100)	
No	1 (3)		-	
How many days since last worked on the street?	23 (77)	60 (1-2920)	11 (100)	7 (1-28)
How often usually sell sex on street?				
Daily	6 (20)		3 (27)	
Weekly	5 (17)		8 (73)	
Less than weekly	16 (53)		-	
Ever used street drugs				
Yes	26 (87)		11 (100)	
No	1 (3)		-	
Ever used heroin	23 (77)		9 (82)	
Days since last used heroin	19 (63)	2 (0-731)	9 (82)	1 (0-6)
Ever used crack cocaine	23 (77)		11(100)	
Days since last used crack cocaine	21 (70)	2 (0-2922)		1 (0-4)
How often use heroin and/or crack cocaine?				
Daily	11 (37)		7 (64)	
Weekly	4 (13)		4 (36)	
Less than weekly	9 (30)		-	
Has an opioid substitute script	13 (43)		6 (55)	
Script type				
Buprenorphine/Subutex®	-		1 (9)	
Methadone	13 (43)		5 (45)	
Used other drugs:				
Alcohol	3 (10)		1 (9)	
Amphetamine	1 (3)		-	
Cannabis	5 (17)		5 (45)	
Spice	2 (7)		-	
MDMA (Ecstasy)	1 (3)			
Tramadol	1 (3)		-	
Sleeping tablets	-		1 (9)	
PCL5 score (possible range 0-80)	-		10 (91)	56 (43-73)

*N and % of those that provided data

For peer review only - http://bmjopen.bmj.com/site/about/guidelines.xhtml

Table 2 Qualitative quotes

Theme/Sub-theme	Quotes
**Recruitment and retention**	
**Acceptability**	*[Face-to-face recruitment] worked well, it wasn't intrusive, you weren't pushy, you know you blended in within the drop-in setting. So I think the women felt that if they did wanna buy into it they would, there was no pressure there. So I think that was done really sensitively.* Service provider 6<br><br>*It [recruitment] was very sort of like confidential and actually it was quite nice 'cause, yeah no one really knew what I was doing when I was doing summut, you know what I mean, which is – like it don't usually happen like that. Everyone knows what I'm doing all the time.* Participant 7
**Improvements**	*I think from a clinical point of view if you remove that criteria [sex work at least once a week in the last calendar month] and then of course there's more chance of getting people through to the finish line to be able to be ready for treatment at the end.* Service provider 4<br><br>*Actively drug using? Yes, that makes sense (…). If they've been able to bring that down themselves maybe another service would be better. Like, what this offered, it's specialistic in this. So if you was able to manage to a level yourself, maybe you don't need [the intervention]… I'm not sure, I think that would be an interesting conversation because if they could bring it down themselves, they'd probably be a lot more stable and a lot more reliable to actually get to the EMDR .* Service provider 7<br><br>*So I think if you were to extend the period of time and say 'Oh actually do you know if you've used within the last three months you can participate in the study and then someone who's three months abstinent or reducing from their street heroin use or their crack use is then exposed to somebody who's going no no no man I'm using up like a party every night'. There'd be that ethical thing within it but it would be nice to see the study opened up to a wider cohort.* Service provider 2
**Facilitators to attendance**	
Encouragement and support to attend	*I would say that I've been quite integral in regards to developing relationships with the women, contacting them for both their individual one to ones and stabilisation groups and also their Thursday DUSSK groups as well. So just keeping that contact going if they were coming in, in our drop-in service I would see them and then sort of give them reminders, did they want little welfare calls, that type of thing.* Service provider 6

For peer review only - http://bmjopen.bmj.com/site/about/guidelines.xhtml

Transport	*It was more of a focus thing where you know she [service provider 6] sort of like coached us as we went down, like you know keeping us like sort of aware of what we've got to be thinking of doing and making sure that, you know, there's nothing wrong.* Participant 7
Food provision	*I was turning up and I was like sort of god like hanging out for (…) that lunch. It was like, not the reason I was turning up but the main reason why I could (…). There is light at the end of the tunnel, you know you're gonna be nourished and fed.' You're gonna be able to concentrate as well.* Participant 7
**Barriers to attendance**	
Unstable lifestyles	*My mental problems, my drug use, everything, just my life, it gets in the way [of attendance].* Participant 1
Mental health	*They're so low resourced, they just don't have the distress tolerance to be able to cope with any more distress, they're already facing so much. Even things like their housing and threats of eviction.* Service provider 8<br><br>*My home life was getting a bit chaotic. My depression was getting really bad as well. So, yeah, and I was waiting for my antidepressants to work but they took a while. Yeah, it was just my depression, that's all.* My anxiety. Participant 6
Sex work	*If I've been working the night before there's no way I could have attended because I'm too tired, because you work all night.* Participant 4
Delays between treatment stages	*It took a little bit of a while and also for them to access their stabilisation groups then their one to ones. I think we may have lost some of the interest.* Service provider 6
**Experience and acceptability of the intervention**	
Initial impressions	*There aren't many services out there, which will offer individual, tailorised counselling and support to the women who have got dual diagnosis and you know mental health, drug misuse. So this study was unique and I think that's what we were all so passionate and so behind it because it was giving the women an opportunity.* Service provider 6
Reason for participating	*I just felt so alone and afraid and stuck and just needed to see if there was some way that I might be able to gain something so – really, if I'm willing to put myself out on the street and sell myself to a complete stranger, knowing that I might die, whatever, so it kind of … I felt I needed to understand why I needed to do this... So it's about me owning my power, and about not letting myself feel as shit about myself as I have done*. Participant 5

For peer review only - http://bmjopen.bmj.com/site/about/guidelines.xhtml

	The post-traumatic stress [treatment] is – is a way of like sort of detoxing your brain. So, you know finding a reason why you do these drugs (…) to like sort of be the reason for me to like say 'Well, I've got to stop now.' You know and get off it. Participant 7
Service providers views on the intervention	*I guess that people thought they weren't going to talk about their traumas [in the drug groups] but if somebody's been raped last night, they're going to need to talk about it, so we were here dealing with that stuff on the spot and then we didn't have no-one to go away and talk about it.* Service provider 10<br><br>*It's very hard to do trauma processing when women to some degree are being traumatised and then having to self-medicate against all of that and then you're trying to work on quite deep attachment developmental trauma stuff from a long time ago. (…) I'd say that trauma processing would be more successful with women who have maybe made a very strong commitment to stop [sex] working.* Service provider 8<br><br>*I would offer it [EMDR] as part of a – as a range of things that are offered…we'd say 'You can have EMDR, trauma focus CBT [Cognitive Behavioural Therapy] or narrative exposure therapy and you'd kind of match the person to what you thought they might be more suited to.* Service provider 8<br><br>*I think they had huge admiration for the workers at [SSW charity], and found them friendly and supportive, but…there wasn't a specific, I don't know, once a month structured 'let's talk about the women and how they've been in the month.* Service provider 9
Participants views of the intervention	*I enjoyed going down there. (...) We had a good laugh and learned something while we were doing it.* Participant 6<br><br>*With it being all woman and not mixed going to (mainstream drug treatment service provider) and doing groups where men are involved is like, I didn't really want to do it but here because it's all women and I know most of the women that come here, we've all been through it, hence why we all come here. So one way or another we've all been through something that we can all relate to.* Participant 3
Intervention characteristics	*It's [SSW charity] familiar and it's comfortable and it's safe.* Service provider 5

For peer review only - http://bmjopen.bmj.com/site/about/guidelines.xhtml

	The groups weren't too big, so you sort of – I knew the people that were coming to the groups which was better, so we'd sort of you know built up a rapport. Participant 4
Impacts of the intervention	*I'm just going to stop [drug use], I'm ready and I'm kind of already preparing for that, so it's kind of brought me to a close, and I mean that as well. Personally it's like, I'm ready, bring it on, I'm like do you know what I've been raped, I've been beaten I've stuck needles in myself(…) I'm done, I'm not playing this game anymore, I deserve better.* Participant 5<br><br>*It's just made (…) me realise I'm not just a, like, drug addict, sex worker. I'm a real person and I've got feelings and, you know, I've got potential. You know, yeah, they [service providers] build me up a lot.* Participant 6<br><br>*When she* [participant 6] *started with [name of intervention] study and she was coming to her Thursday [drug] groups, she didn't want to be associated with street sex working. So she said 'Can you call me (own name rather than working name)?' I could have cried (…). She was owning her own name and taking back ownership of who she is rather than somebody who was street sex working.* Service provider 6<br><br>*Their chaoticness. (…) To manage that in a [mainstream drug service] group setting would be difficult and I'm not sure how they would manage that. I just know how much regularly how they've turned up [to the intervention drug treatment groups] chaotic and they've turned up leaking out trauma. … I'm far from confident that they would be able to sit under them [mainstream drug service] rules enough to be a part of what it is for here[research study], due to the level of flexibility here and that they would be able to talk about what their problem is without mentioning what they do and that might make them vulnerable* Service provider 7
Fidelity	*I guess we were kind of thinking of it in a really linear sense, that the women would engage in the drug groups and then reduce their drug use to then move on to the next group and I'm not sure that that actually happened in reality.*<br>Service provider 5<br><br>*In the beginning we went in doing the same sort of work that we would do here [mainstream drug services], and it's getting them to look at their behaviour, and the consequences of it and stuff, and it didn't work with these women, it's too much, too direct.* Service provider 10

For peer review only - http://bmjopen.bmj.com/site/about/guidelines.xhtml

Table 3 Attendance and retention of participants (top four rows – those that attended trauma screening)

	Open group Getting started			Attended Trauma Screening	Closed group Getting started			5 mandatory Stabilisation group sessions		One-to-one sessions				Referred to which services:
Participant	Eligible sessions	Attended sessions*	% Attended		Eligible sessions	Attended sessions	% Attended	Attended sessions	% Attended	Treatment	Eligible sessions**	Attended sessions*	% Attended	
1	28	10	36	Yes	26	4	15	3	60	Ext. stabilisation	8[3]	1 (1)	13	Mental health
2	27	8	30	Yes	26	11	42	5	100	EMDR	12[3]	4 (1) $	33	Mental health
3	20	7 (2)	35	Yes	26	3	12	1	20	Ext. stabilisation	8[4]	0	0	Mental health
4	25	19 (5)	76	Yes	26	15	58	4	80	EMDR	12[2]	8 (4) $	67	Mental health & mainstream drug
5	28	5	18	No										
6	28	1 (1)	4	NA										
7	25	3	12	NA										
8	23	0	0	NA										
9	22	0	0	NA										
10	19	0	0	NA										
11	19	0	0	NA										
Total		53		4		33		13				13		

*N session participant arrived late/left early indicated in superscript round brackets; **N sessions cancelled due to non-attendance in square brackets; NA=Not actively invited to sessions; $ Includes one review session; Ext. stabilisation=Extended stabilisation, EMDR=Eye movement desensitization and reprocessing therapy

For peer review only

Table 4 Health Economics

	1. Getting started		2. Trauma screening		3. Stabilisation group		4. One-to-one sessions	
**Service description**								
Session lengths - range	90 - 120 minutes [1]		60 minutes		60 minutes		60 - 90 minutes [2]	
Number of sessions held - total	52		8		5		28	
Eligible participants[3] - total	n = 11		n = 5[4]		n = 4		n = 4	
Attendees per session - range	0 - 4		1		2 - 4		1	
**Costs**	**Sub-total £**	**£ per ppt**	**Sub-total £**	**£ per ppt**	**Sub-total £**	**£ per ppt**	**Sub-total £**	**£ per ppt**
**A. Staff**								
Service manager	£1,359.76	£123.61	£95.09	£19.02	£47.54	£11.89	£266.25	£66.56
Drug group facilitators[5]	£3,000.20	£272.75	£0.00	£0.00	£0.00	£0.00	£0.00	£0.00
Art worker	£123.98	£11.27	£0.00	£0.00	£0.00	£0.00	£0.00	£0.00
Clinical Psychologist	£0.00	£0.00	£636.00	£127.20	£600.00	£150.00	£2,040.00	£510.00
**B. Facilities**								
Space rental	£1,270.50	£115.50	£224.00	£44.80	£140.00	£35.00	£574.00	£143.50
**C. Travel**								
Transporting materials	£73.78	£8.44	£0.00	£0.00	£11.90	£2.98	£0.00	£0.00
Car lifts for service-users (petrol)	£38.72	£3.52	£2.24	£0.45	£3.36	£0.84	£1.20	£0.30
Public transport for participants	£70.20	£6.38	£0.00	£0.00	£3.90	£0.98	£3.90	£0.98
Taxis for participants	£55.00	£5.00	£0.00	£0.00	£62.20	£15.55	£11.00	£2.75
**D. Materials**								
Art supplies	£30.00	£2.73	£0.00	£0.00	£0.00	£0.00	£0.00	£0.00
Stationary	£0.00	£0.00	£0.00	£0.00	£5.00	£1.25	£0.00	£0.00
Voucher incentives	£0.00	£0.00	£0.00	£0.00	£130.00	£32.50	£0.00	£0.00
Refreshments[6]	£820.00	£74.55	£0.00	£0.00	£10.00	£.2.50	£0.00	£0.00
**Summary**								
Total cost	£6,842.13	£622.01	£957.33	£191.47	£1,013.90[7]	£253.48	£2,896.35	£724.09
Total cost per session	£131.58	£11.96	£119.67	£23.93	£202.78	£50.70	£103.44	£25.86

Note: 1 = Sessions were originally were 90 minutes, however when sandwiches were provided drug group facilitators arrived 30 minutes prior to session to be with participants while they ate, 2 = One-to-one EMDR sessions were 90 minutes and one-to-one Stabilisation sessions were 60 minutes, 3 = participants, 4 = Five participants were eligible for screening, however only four participants were successfully screened, 5 = Getting started groups were facilitated by two drug group facilitators, and 6 = Sandwiches and biscuits, 7 = Total cost without vouchers would have been £883.90 .

**Heat map description:**	**£0.00**	**£0.01 - £99.99**	**£100 - £499.99**	**£500.00 - £1999.99**	**£2000.00 +**

For peer review only - http://bmjopen.bmj.com/site/about/guidelines.xhtml

Figure legend

Figure displaying the flow and numbers of potential and consented participants through the DUSSK study. Numbers may include more than one approach to potential participants by different recruiters. Includes reasons provided by women spoken to, but not screened, those screened but not eligible/ not consented and consented participants who did not reach the 'getting started' closed group intervention stage.

For peer review only

For peer review only - http://bmjopen.bmj.com/site/about/guidelines.xhtml

Figure 1: Flow of participants through the DUSSK study

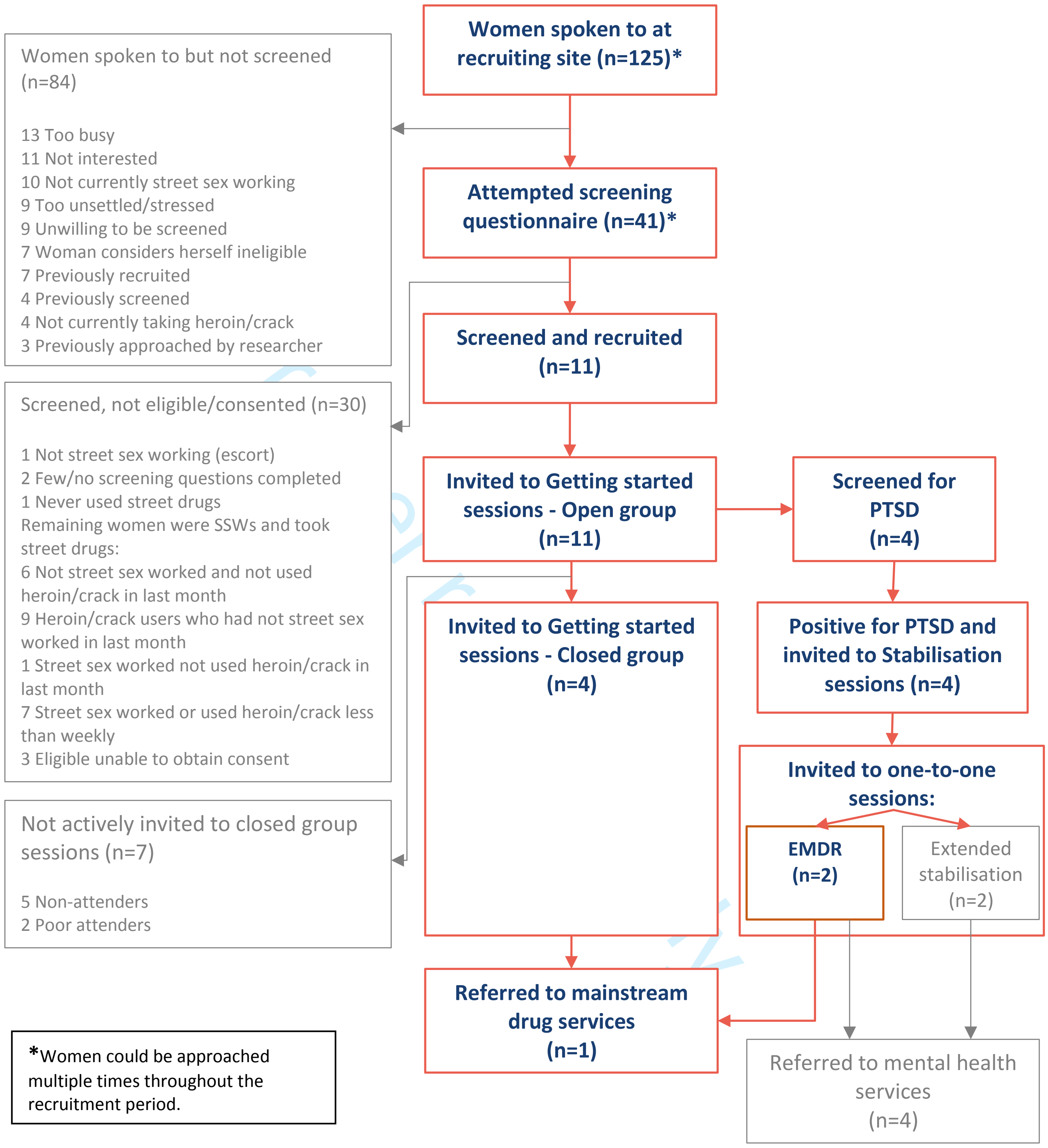

Reporting checklist for cohort study.

Based on the STROBE cohort guidelines.

Instructions to authors

Complete this checklist by entering the page numbers from your manuscript where readers will find each of the items listed below.

Your article may not currently address all the items on the checklist. Please modify your text to include the missing information. If you are certain that an item does not apply, please write "n/a" and provide a short explanation.

Upload your completed checklist as an extra file when you submit to a journal.

In your methods section, say that you used the STROBE cohortreporting guidelines, and cite them as:

von Elm E, Altman DG, Egger M, Pocock SJ, Gotzsche PC, Vandenbroucke JP. The Strengthening the Reporting of Observational Studies in Epidemiology (STROBE) Statement: guidelines for reporting observational studies.

		Reporting Item	Page Number
**Title and abstract**			
Title	#1a	Indicate the study's design with a commonly used term in the title or the abstract	1
Abstract	#1b	Provide in the abstract an informative and balanced summary of what was done and what was found	2
**Introduction**			
Background / rationale	#2	Explain the scientific background and rationale for the investigation being reported	4
Objectives	#3	State specific objectives, including any prespecified hypotheses	4
**Methods**			

For peer review only

Study design	#4	Present key elements of study design early in the paper	5
Setting	#5	Describe the setting, locations, and relevant dates, including periods of recruitment, exposure, follow-up, and data collection	5
Eligibility criteria	#6a	Give the eligibility criteria, and the sources and methods of selection of participants. Describe methods of follow-up.	5
Eligibility criteria	#6b	For matched studies, give matching criteria and number of exposed and unexposed	N/A
Variables	#7	Clearly define all outcomes, exposures, predictors, potential confounders, and effect modifiers. Give diagnostic criteria, if applicable	8
Data sources / measurement	#8	For each variable of interest give sources of data and details of methods of assessment (measurement). Describe comparability of assessment methods if there is more than one group. Give information separately for for exposed and unexposed groups if applicable.	7
Bias	#9	Describe any efforts to address potential sources of bias	N/A
Study size	#10	Explain how the study size was arrived at	7
Quantitative variables	#11	Explain how quantitative variables were handled in the analyses. If applicable, describe which groupings were chosen, and why	7
Statistical methods	#12a	Describe all statistical methods, including those used to control for confounding	8
Statistical methods	#12b	Describe any methods used to examine subgroups and interactions	N/A
Statistical methods	#12c	Explain how missing data were addressed	N/A
Statistical methods	#12d	If applicable, explain how loss to follow-up was addressed	N/A
Statistical methods	#12e	Describe any sensitivity analyses	N/A

For peer review only

Results

Participants	#13a	Report numbers of individuals at each stage of study—eg numbers potentially eligible, examined for eligibility, confirmed eligible, included in the study, completing follow-up, and analysed. Give information separately for for exposed and unexposed groups if applicable.	20
Participants	#13b	Give reasons for non-participation at each stage	9
Participants	#13c	Consider use of a flow diagram	20
Descriptive data	#14a	Give characteristics of study participants (eg demographic, clinical, social) and information on exposures and potential confounders. Give information separately for exposed and unexposed groups if applicable.	9
Descriptive data	#14b	Indicate number of participants with missing data for each variable of interest	21
Descriptive data	#14c	Summarise follow-up time (eg, average and total amount)	26
Outcome data	#15	Report numbers of outcome events or summary measures over time. Give information separately for exposed and unexposed groups if applicable.	9
Main results	#16a	Give unadjusted estimates and, if applicable, confounder-adjusted estimates and their precision (eg, 95% confidence interval). Make clear which confounders were adjusted for and why they were included	9-12
Main results	#16b	Report category boundaries when continuous variables were categorized	N/A
Main results	#16c	If relevant, consider translating estimates of relative risk into absolute risk for a meaningful time period	N/A
Other analyses	#17	Report other analyses done—e.g., analyses of subgroups and interactions, and sensitivity analyses	9-12

Discussion

Key results	#18	Summarise key results with reference to study objectives	13

For peer review only

Limitations	#19	Discuss limitations of the study, taking into account sources of potential bias or imprecision. Discuss both direction and magnitude of any potential bias.	13
Interpretation	#20	Give a cautious overall interpretation considering objectives, limitations, multiplicity of analyses, results from similar studies, and other relevant evidence.	15
Generalisability	#21	Discuss the generalisability (external validity) of the study results	N/A
**Other Information**			
Funding	#22	Give the source of funding and the role of the funders for the present study and, if applicable, for the original study on which the present article is based	16

The STROBE checklist is distributed under the terms of the Creative Commons Attribution License CC-BY. This checklist was completed on 16. December 2019 using https://www.goodreports.org/, a tool made by the EQUATOR Network in collaboration with Penelope.ai

For peer review only
